# Rehabilitation enhances epothilone-induced locomotor recovery after spinal cord injury

**DOI:** 10.1093/braincomms/fcad005

**Published:** 2023-01-13

**Authors:** Jarred M Griffin, Sonia Hingorani Jai Prakash, Till Bockemühl, Jessica M Benner, Barbara Schaffran, Victoria Moreno-Manzano, Ansgar Büschges, Frank Bradke

**Affiliations:** Laboratory for Axonal Growth and Regeneration, German Center for Neurodegenerative Diseases (DZNE), Bonn 53127, Germany; Neuronal and Tissue Regeneration Laboratory, Centro de Investigación Príncipe Felipe (CIPF), Valencia 46012, Spain; Department of Animal Physiology, Institute of Zoology, University of Cologne, Cologne 50674, Germany; Laboratory for Axonal Growth and Regeneration, German Center for Neurodegenerative Diseases (DZNE), Bonn 53127, Germany; Laboratory for Axonal Growth and Regeneration, German Center for Neurodegenerative Diseases (DZNE), Bonn 53127, Germany; Neuronal and Tissue Regeneration Laboratory, Centro de Investigación Príncipe Felipe (CIPF), Valencia 46012, Spain; Department of Animal Physiology, Institute of Zoology, University of Cologne, Cologne 50674, Germany; Laboratory for Axonal Growth and Regeneration, German Center for Neurodegenerative Diseases (DZNE), Bonn 53127, Germany

**Keywords:** spinal cord injury, epothilone, rehabilitation, axon regeneration, neuroplasticity

## Abstract

Microtubule stabilization through epothilones is a promising preclinical therapy for functional recovery following spinal cord injury that stimulates axon regeneration, reduces growth-inhibitory molecule deposition and promotes functional improvements. Rehabilitation therapy is the only clinically validated approach to promote functional improvements following spinal cord injury. However, whether microtubule stabilization can augment the beneficial effects of rehabilitation therapy or act in concert with it to further promote repair remains unknown. Here, we investigated the pharmacokinetic, histological and functional efficacies of epothilone D, epothilone B and ixabepilone alone or in combination with rehabilitation following a moderate contusive spinal cord injury. Pharmacokinetic analysis revealed that ixabepilone only weakly crossed the blood–brain barrier and was subsequently excluded from further investigations. In contrast, epothilones B and D rapidly distributed to CNS compartments displaying similar profiles after either subcutaneous or intraperitoneal injections. Following injury and subcutaneous administration of epothilone B or D, rats were subjected to 7 weeks of sequential bipedal and quadrupedal training. For all outcome measures, epothilone B was efficacious compared with epothilone D. Specifically, epothilone B decreased fibrotic scaring which was associated with a retention of fibronectin localized to perivascular cells in sections distal to the lesion. This corresponded to a decreased number of cells present within the intralesional space, resulting in less axons within the lesion. Instead, epothilone B increased serotonergic fibre regeneration and vesicular glutamate transporter 1 expression caudal to the lesion, which was not affected by rehabilitation. Multiparametric behavioural analyses consisting of open-field locomotor scoring, horizontal ladder, catwalk gait analysis and hindlimb kinematics revealed that rehabilitation and epothilone B both improved several aspects of locomotion. Specifically, rehabilitation improved open-field locomotor and ladder scores, as well as improving the gait parameters of limb coupling, limb support, stride length and limb speed; epothilone B improved these same gait parameters but also hindlimb kinematic profiles. Functional improvements by epothilone B and rehabilitation acted complementarily on gait parameters leading to an enhanced recovery in the combination group. As a result, principal component analysis of gait showed the greatest improvement in the epothilone B plus rehabilitation group. Thus, these results support the combination of epothilone B with rehabilitation in a clinical setting.

## Teaser

Using a moderate spinal cord injury paradigm combined with histological analyses and multiplexed behavioural analyses of gait, Griffin *et al.* show that pharmacological microtubule stabilization and rehabilitation act on complementary aspects of locomotion to enhance functional recovery.

## Graphical Abstract

**Graphical abstract:**
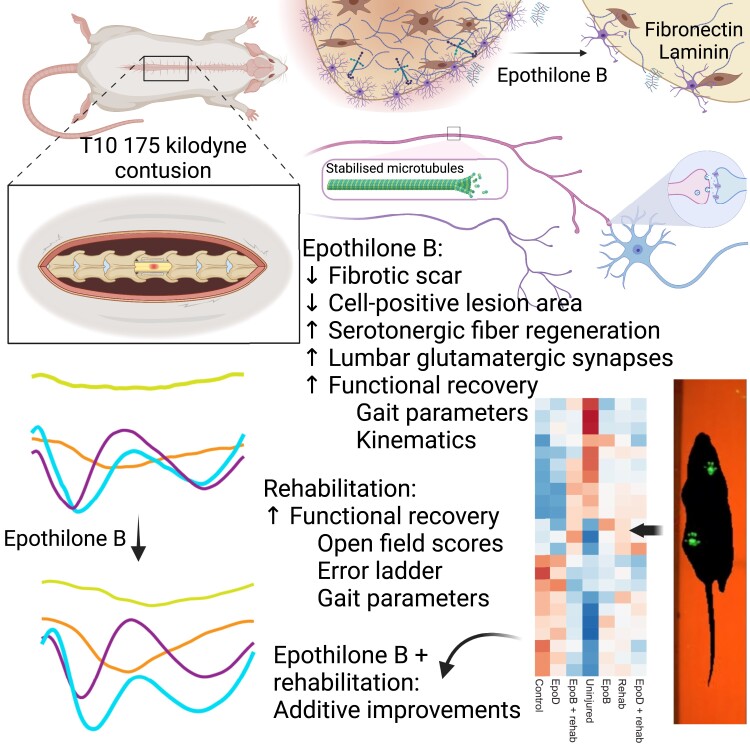

## Introduction

Lesioned axons of the adult CNS fail to regrow, limiting recovery after spinal cord injury (SCI). This is due to the poor intrinsic regenerative capacity of adult neurons,^1^ as well as extrinsic inhibitory factors, including myelin-associated molecules and chondroitin sulphate proteoglycans (CSPGs).^2,3^ To date, there is no regenerative therapy established to treat SCI patients. However, several preclinical interventions overcome these obstacles, including removal of inhibitory molecules, the addition of growth-permissive cell or tissue transplantation, provisions of trophic support and manipulation of pro-regenerative neuronal signalling pathways.^4–10^ Regardless of these advancements, it is becoming apparent that singularly they offer only a limited improvement in experimental animals and are unlikely to facilitate meaningful functional regeneration in humans.^11,12^ Utilizing appropriate rehabilitation regimes is a promising avenue for shaping new circuits created by regeneration-promoting therapies into functional connections in preclinical studies.^13–16^ Likewise, clinical evidence highlights that rehabilitative strategies improve function in individuals with SCI,^17^ creating optimism for clinical trials that utilize complementary therapeutics.

The neuronal cytoskeleton determines the regenerative response of lesioned axons.^18–21^ Disorganized microtubules underlie the formation of retraction bulbs, with the stabilization of microtubules coaxing growth cone formation and protrusion.^22,23^ Microtubule stabilization also restricts the polarization and migration of scar-forming fibroblasts.^24^ Systemic administration of the microtubule stabilizing drugs epothilone D (epoD) or B (epoB) results in adequate CNS penetration and distribution, reduction of fibrotic scarring and deposition of inhibitory CSPGs, reduced axon dieback, induced axonal growth and improved motor functions after mild SCI and stroke.^24–27^ While the mechanisms are poorly understood, rehabilitation can remodel spinal cord axonal connections underlying motor recovery.^28^ We, therefore, hypothesized that epothilones and rehabilitation could act additively to improve functional outcomes.

EpoD and epoB display different pharmacokinetic (PK) profiles, whereby epoB persists in the CNS compartment for much longer than epoD and has been proposed to be more efficacious.^25,26^ However, a direct comparison of these two drugs following SCI is still lacking. Given the promising results of epothilones following a mild 150 kdyn contusion,^24–26^ we investigated their effects in a more severe contusion injury of 175 kdyn, with and without rehabilitation.

Here we show that epoB has distinct histological effects on spinal cord injured rats that are not elicited by rehabilitation, yet both interventions individually improve various aspects of locomotor recovery. Consequently, their combination resulted in functional recovery unmet by the singular components. Thus, this combinatorial treatment provides a key step towards a clinical evaluation of rehabilitation assessment, together with pharmacological microtubule stabilization.

## Materials and methods

### Animals

All animal experiments were performed in accordance with the Animal Welfare Act and the guidelines of the North Rhine-Westphalia State Environment Agency (Landesamt für Natur, Umwelt und Verbraucherschutz). Female Sprague–Dawley rats (Janvier Labs) weighing between 220 and 250 g were used throughout the study. A total of 194 rats were used in this study including those used for the PK study. The animals were housed three rats per cage with room temperature controlled at 21–22°C, and an artificial 12 h light/dark cycle. Rats were given food and water ad libitum throughout the experiment. Methods and results are written in accordance with the ARRIVE (Animal Research: Reporting of In Vivo Experiments) guidelines for publishing *in vivo* research.^29^

### PK analysis

The PK study was conducted by Pharmacelsus GmbH. These experimental procedures were approved by and conducted in accordance with the regulations of the local Animal Welfare authorities (Landesamt für Gesundheit und Verbraucherschutz, Abteilung Lebensmittel- und Veterinärwesen, Saarbrücken). Rats were injected subcutaneously (s.c.) or intraperitoneally (i.p.) with epoD (1.5 mg/kg; Abcam, ab143616), epoB (0.75 mg/kg; Selleck Chemicals S1364) or ixabepilone (0.75 or 1.5 mg/kg; Ixempra Cay23732-10). Drugs were diluted in 50% dimethyl sulfoxide (DMSO)/saline. Animals were euthanized at 6 h, 1, 7 or 14 days after injections and blood plasma and spinal cord tissue samples were collected. Three animals per time point and group were used, for a total of 72 used in the PK study. Drug concentrations in the samples were quantified by liquid chromatography–mass spectrometry (LC–MS) using an Accela 1250 UHPLC (Ultra High Performance Liquid Chromatography) system, Accela Open Autosampler and Q-Exactive mass spectrometer (Thermo Fisher Scientific).

### Surgical procedures and treatment

Rats were administered with analgesics and antibiotics (Meloxicam, 1 mg/kg, s.c.; Enrofloxacin, 5 mg/kg, s.c.) and anaesthetized with isoflurane. Skin was shaved and cleaned with ethanol and iodine swabs, sequentially. A laminectomy was performed to expose the spinal cord at spinal level T10 and impacted at the midline with a force of 175 kdyn using an Infinite Horizon Impactor (Precision Systems Instrumentation). Following the impaction, overlying musculature and skin were sutured. Two millilitres of 5% glucose (Braun) were administered s.c. and animals were transferred to a heated cage to recover for at least an hour before being returned to a fresh home cage. Animals were administered antibiotics (Enrofloxacin s.c.) and analgesics (Meloxicam, Buprenorphine 0.05 mg/kg; s.c.) for 3 days following the surgery. Bladders were expressed manually three times daily until the normal voiding response returned. Following the dosing scheme of our previous studies,^24–26^ animals were randomly allocated to treatment groups and received blinded s.c. injections of either epoD (1.5 mg/kg; Abcam, ab143616), epoB (0.75 mg/kg; Selleck Chemicals S1364) or vehicle (50% DMSO) on Days 1 and 15 post-injury.

#### Rehabilitation

Hindlimb bipedal and quadrupedal exercise training was initiated 3 weeks after the injury (1 week after the second administration of epoD/epoB). This blend of training was selected to reinforce supraspinal-controlled initiation and modulation of bipedal locomotion as well as the reorganization and re-engagement of rostrocaudal spinal interneuron networks that occurs with quadrupedal training. A custom-adapted five-lane rodent treadmill (Pan Lab/Harvard Apparatus, #LE8710RTS) was used and animals were supported upright in harnesses and positioned so the hind paws touched the surface of the treadmill as previously reported.^30^ This apparatus and harness allowed for animals to be kept upright whilst having near full weight-bearing movements on their hindlimbs. Training beginning 1 week prior to the injury involved habituation of the rats to the treadmill and the suspension harnesses for 3 days followed by guiding them to walk on their hindlimbs for 30 min each for 3 days. By 3 weeks post-SCI, all of the animals recovered at least weight-supported planter stepping and therefore were able to be trained effectively for bipedal stepping. The rats completed bipedal training at a speed of 20–35 cm/s for 20 min a day, 5 days a week, for 7 weeks in total. Immediately following completion of bipedal training sessions, the rats underwent 20 min of quadrupedal exercise training at a speed of 30–40 cm/s.

### Tissue processing and immunohistochemistry

Ten weeks after the injury and following the final behaviour tests, animals were overdosed with ketamine/xylazine (100/10 mg/kg, i.p.) and transcardially perfused with 0.9% saline followed by 4% paraformaldehyde in 0.1 M phosphate buffer. The spinal cords were removed and post-fixed overnight at 4°C before being cryoprotected in 30% sucrose. Two regions of interest were dissected. The first encompassed one centimetre of the spinal cord with the lesion at the centre. The second region encompassed 0.5 cm of tissue caudal to the first region. This second region represents L1–L4 spinal cord level and was used for investigating lumbar plasticity. Both pieces of tissue were cryosectioned at 30 µm, mounted onto positively charged slides (SuperFrost Plus; Fisher Scientific) and arranged so that each section was 600 µm apart. Slides were then frozen until required. Immunohistochemistry (IHC) was performed to investigate lesion size, potential neuroprotection, fibrotic scarring, blood vessels, serotonergic fibres and glutamatergic vesicles. Briefly, slides were washed with PBST (Phosphate Buffered Saline with 0.2% Triton X-100) three times for 10 min each, followed by blocking with 10% normal goat or donkey serum. Primary antibodies (Supplementary Table 1) were diluted in PBST and added to the slides at room temperature overnight. The slides were washed with PBST three times for 10 min each before addition of the appropriate secondary antibodies and 4′,6-diamidino-2-phenylindole (DAPI; Invitrogen; 1:10 000) at room temperature for 2 hours (Supplementary Table 1). The slides were then washed three times with PBS (Phosphate Buffered Saline) before being coverslipped with Fluoromount (ThermoFischer).

### Microscopy and image analyses

Tile-scanned images of immuno-stained whole coronal spinal cord sections were obtained using a Zeiss Axioscan.Z1 slide scanner microscope at × 10 or × 20 magnification. This microscope was used to ensure that acquisition was automated and that the parameters were maintained for each IHC analysis. All image analyses were done blinded to the treatment groups.

#### Injury site analyses

Injury size and tissue sparing were determined by measuring the area delineated by glial fibrillary acidic protein (GFAP) immunolabeling in Zen Blue software and then subtracting from the total tissue circumference. All remaining image analysis was performed in ImageJ. The number of neuronal-nuclei (NeuN)-positive neuronal nuclei over the injury was calculated by first thresholding images to positive staining for NeuN and then converting the images to binary. To count the number of NeuN-positive nuclei, the watershed filter was applied and particles were measured within the spinal cord parenchyma measuring between 20 and 2400 μm^2^. For the laminin and fibronectin markers of fibrotic scaring analysis, a threshold was applied to positive staining, of which the area within the spinal cord parenchyma tissue was measured. To investigate the cell-positive lesion area and neuronal expression within the lesion, we stained for GFAP, β-tubulin-III (βtubIII) and DAPI. Sections representing the lesion epicentre, 600 µm rostral to the lesion and 600 µm caudal to the lesion were selected. The lesion border was delineated by GFAP staining to determine what we term the ‘intralesional space’. Within the intralesional space, we measured cell number as well as cell-positive and cell-negative (cystic cavitation) areas. The number of cells was counted using particle analysis and the area of βtubIII-positive immunoreactivity was measured. For serotonin (5-HT) analysis, three transverse sections 1800–3000 µm caudal to the lesion per animal were analysed. Regions of interest were selected covering an area comprising the ventral horns. These regions were assigned a pixel threshold representative of positive immunostaining that was maintained across all analyses, whereby pixel area above this threshold was summed and averaged across each animal in each experimental group.

#### Lumbar site analysis

We performed IHC probing for vesicular glutamate transporter 1 (VGluT1) on coronal sections of lumbar spinal cord spanning L1–L4, as detailed above. Images were captured at ×20 magnification on an AxioscanZ1 microscope, and then converted to 8-bit before thresholding for positive staining. Integrated density and area of fluorescence were measured in Laminae I–II, III–V and then VI–X as determined by the atlas of the rat lumbar spinal cord. Eight sections spanning the lumbar cord were analysed per animal. Data were represented as integrated density of staining per area.

### Behavioural functional assessments

#### Basso, Beattie and Bresnahan scoring and horizontal ladder test

One week prior to injury, all animals were habituated and trained to complete each behaviour task for 30 min daily over 3 days. Basso, Beattie and Bresnahan locomotor scale (BBB) scoring was carried out by two blinded observers in a circular open field for 5 min each as previously described.^30,31^ For the horizontal ladder test, a 100 cm ladder with irregularly spaced rungs was used. Each animal completed three runs of the ladder while being recorded using a GoPro Hero5 camera. The footage was analysed frame-by-frame by a blinded observer to capture footfalls and total steps. Steps were considered an error if the paw slipped from the rung, and both partial and full slips were counted. The average number of errors was recorded over the three runs and expressed as a percentage of the total steps.

#### Two-dimensional kinematic recordings and analysis

Data acquisition and kinematic analysis using the MotoRater System (TSE Systems, Germany; #E-303030-RM) was conducted as previously described.^32^ The hindquarters of the rats were shaved, and markings were placed on the following landmarks of the hindlimbs: iliac crest (IC), greater trochanter (hip), lateral malleolus (ankle), lateral epicondyle (knee) and the metatarsophalangeal joint of fifth toe. Videos were recorded of over-ground walking from left and right lateral views using a Matrix Vision mvBlueFOX3 CMOS (Complementary Metal-Oxide Semiconductor) camera at a resolution of 1496 × 642 pixels and a frame rate of 193 frames/s. At least five passes of the runway were recorded for each animal. The markers were then tracked in the videos using TSE Motion V9.2.2 (Simi Reality Motion Systems GmbH) pattern recognition software. This resulted in *x*- and *y*-coordinates for all markers on both body sides over the time course of the runs. Based on these marker positions, we calculated four joint angle time courses for each hind leg and each time point in all trials. These were defined as the angle between the horizontal axis of the video and the IC-hip segment (IC angle), the angle between the IC-hip and the femur (hip joint), the angle between the femur and the tibia (knee joint) and the angle between the tibia and the ankle-toe segment (ankle joint). To average all steps in a particular leg, we calculated the movement speed of the foot markers as a proxy for the onset of individual steps: whenever the speed of a foot marker dropped below an empirically determined threshold, the leg was considered to have initiated a stance movement. Complete step cycles were defined as the movement from a lift-off event to the next. For further analysis, we only considered complete step cycles. After normalizing each individual step cycle to 100 time points, the joint angle time courses associated with these step cycles were averaged per body side (i.e. left and right legs were analysed separately). We further calculated two additional kinematic measures: the average normalized IC joint height (defined by the average distance of this marker to the floor of the walkway during individual trials normalized to the uninjured condition), and the maximum amplitudes for all joint angles (defined by the absolute difference between its maximum and minimum in an individual’s average joint angle time course).

#### Gait analysis

The CatWalk XT gait analysis system (Noldus)^33,34^ was used to quantitate parameters of gait and locomotion in the rats 1 week prior to their injury until 10 weeks post-injury. Camera and recording settings involved a green detection threshold of 0.10, camera gain of 11.60 dB, red ceiling light voltage of 18.0 and green walkway light voltage of 19.0. For a run to be considered successful, there had to be a maximum run variation of <60% and completion within 5 s. Four successful runs per animal were recorded. Every run was first classified by the automated footprint recognition of the CatWalk XT software. Each run was then manually reviewed and its annotation corrected as needed by a blinded investigator. Gait parameters are represented as an average of the four runs per animal, per time point. A total of 37 parameters were used for analysis (Supplementary Table 2). Parameters were assigned classification and sub-classifications, and the data set was then pooled for like-parameters, i.e. left and right hindpaw print area was pooled into the one parameter of ‘hindpaw print area’. This produced 23 parameters used for principal component analysis (PCA). First, the data for each animal were averaged and each averaged data set of 23 parameters was treated as an entry in the data matrix for PCA, resulting in a 23-by-23 matrix. Rows indicated individual animals, and columns indicated the different parameters. This data set was centred and each column normalized to a standard deviation of 1 before calculating PCA. We extracted the explained variance for each principal component (PC) and the coefficients for the first three PCs. Following this, we processed this PCA data set by collapsing columns with similar annotations to the means and performed heatmap hierarchical clustering to identify correlation between groups.

### Data exclusion and statistical analyses

An *a priori* sample size calculation was conducted based on the mean and standard deviation of our previous behaviour results, using an *α* of 0.02 and a 1 − *β* of 0.95.^30^ The calculation returned a minimum number of 14 animals per group. An exclusion criterion was set prior to the initiation of the study, accounting for if the combined hindlimb BBB score of an animal post-surgery was greater than five at day 3 after injury; if BBB scores did not improve past a combined score of nine by the end of the 3 weeks (due to inability to complete the ladder task); and if the animal was euthanized because of complications of the surgery, as measured by outcomes such as incontinence lasting longer than 2 weeks or automutilation. Due to government restrictions during the COVID pandemic, animals that were initially planned to be in a rehabilitation group were forced to be re-allocated to control and epoD groups, resulting in a higher sample number within these groups.

All numerical values are reported as means ± standard error of the mean (SEM) unless otherwise stated. Statistical analyses were performed using GraphPad Prism 9. To assess whether data sets followed a normal distribution, a D’Agostino and Pearson normality test with *α* = 0.05 was applied to check for Gaussian distribution. Data sets passed this test and as such, parametric one-way and two-way ANOVA statistical analyses were applied. The exception to this was the statistical analysis for the kinematics data which utilized the Wilcoxon rank-sum test. All statistics and *post hoc* tests for multiple comparison corrections are stated in the text where appropriate. For all analyses: *P < 0.05, **P < 0.001, ***P < 0.0001.

## Results

### Ixabepilone fails to cross the blood–brain barrier, whilst epothilone D and B rapidly distribute following intraperitoneal or subcutaneous injections

**Figure 1:**
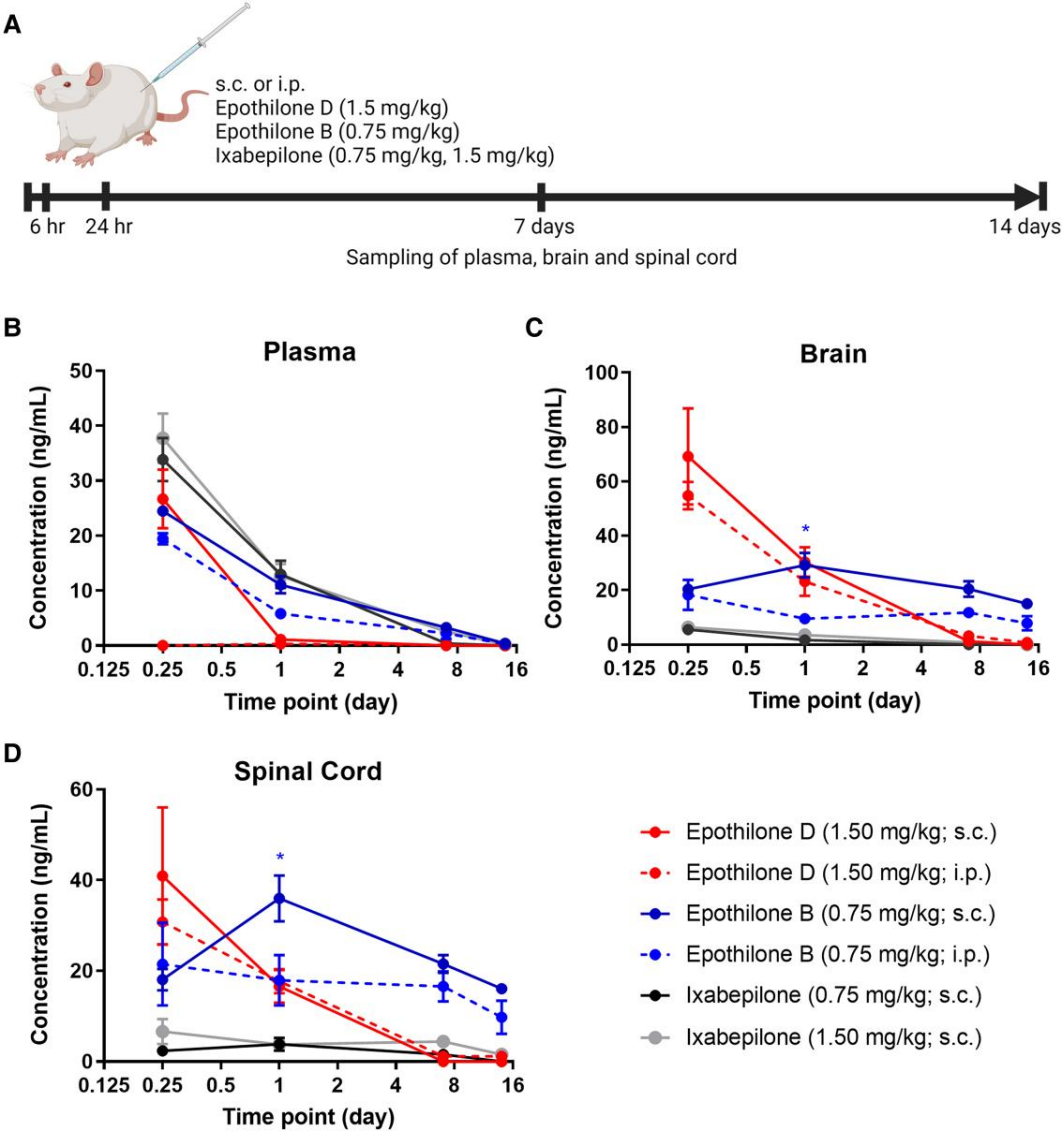
**PK profiles of epoD, epoB and ixabepilone following subcutaneous or i.p. injection**. (**A**) Timeline of epothilone injections and tissue collection. (**B–D**) Concentration profiles of epothilones in plasma, brain and spinal cord tissue quantified by LC–MS. Created with BioRender.com. Plotted data are the mean drug concentration ±SEM. *P < 0.05 by two-way ANOVA and Bonferroni *post hoc* (asterisks compares the epoB groups). *n* = 3 animals per group and time point.

Our previous studies established the dosing schemes of i.p. injections of epoB and D based on PKs and the pharmacodynamics of tubulin acetylation and tyrosination.^24–26^ In general, absorption of lipophilic drugs occurs slower through the s.c. route and can be advantageous when increased presence of the drug is desired.^35^ Given the lipophilicity of epothilones,^36^ we tested whether s.c. administration of epothilones displays a PK profile similar to those for i.p. injection.^24,25^ A pilot study showed that naïve and contused adult animals displayed comparable concentrations of epoD in the CNS after i.p. injections 1 or 3 days post-injury (Supplementary Fig. 1). We, therefore, focused our PK analysis on naïve adult animals (Fig. 1A). In addition, we tested the PK of a third epothilone, ixabepilone, which is to date the sole drug from the epothilone family with FDA (Food and Drug Administration) approval for use in humans. LC–MS of biological extracts, however, showed that ixabepilone is only weakly BBB-permeable, with minimal concentrations of the drug detected in the brain and spinal cord tissue (Fig. 1B–D). Hence, ixabepilone was subsequently not considered a potential systemic treatment for SCI, excluding it from the remainder of this study. In contrast, epoD and epoB rapidly distributed from the plasma to the CNS (Fig. 1B–D). However, these epothilones showed differing retention rates in the CNS: concentrations of epoD were undetectable in CNS tissue 7 days after injection, whereas epoB persisted for up to 14 days (Fig. 1C and D). Notably, s.c. injections of epoB resulted in greater drug concentrations in the CNS 24 hours after injections compared with i.p. (Fig. 1C and D; *P* < 0.05; two-way ANOVA, Bonferroni *post hoc*). Therefore, all the epothilones were administered s.c. in the following experiments.

### Neuroprotection is not conferred by epothilones or rehabilitation

**Figure 2:**
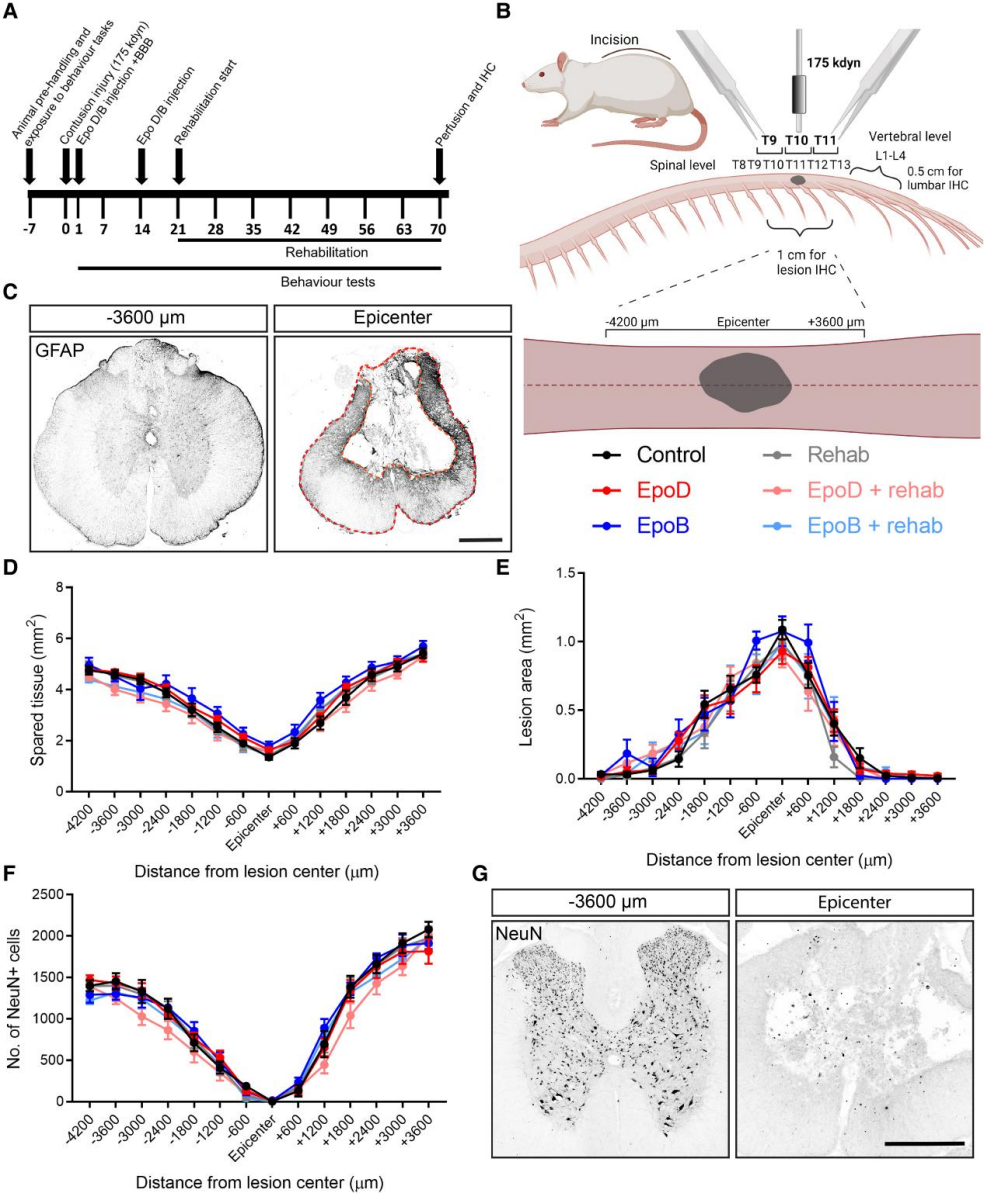
**Neither epothilones nor rehabilitation confers neuroprotection**. (**A**) Scheme of the study design displaying the timing of injury, rehabilitation and behavioural tests. (**B**) Scheme depicting the location of the contusive injury and what regions of the spinal cord were sectioned for IHC. Created with BioRender.com. (**C**) Representative fluorescence images of GFAP-labelled spinal cord sections at the lesion epicentre or −3600 µm rostral demarcating the lesion area and spared tissue (orange dashed lines; red dashed lines show the tissue border; control animal; pixel values inverted; scale bar = 500 µm). (**D** and **E**) Quantification of lesion spared tissue and lesion size throughout the lesioned spinal cord determined by GFAP lesion demarcation (control *n* = 16; epoD *n* = 18; epoB *n* = 11; rehabilitation *n* = 10; epoD + rehabilitation *n* = 12; epoB + rehabilitation *n* = 12). (**F**) Quantification of the number of neurons throughout the lesioned spinal cord determined by counting NeuN immunolabelling (control *n* = 13; epoD *n* = 13; epoB *n* = 12; rehabilitation *n* = 10; epoD + rehabilitation *n* = 12; epoB + rehabilitation *n* = 11). (**G**) Representative fluorescence images of NeuN-labelled spinal cord sections at the lesion epicentre or −3600 µm rostral (control animal; pixel values inverted; scale bar = 500 µm). Plotted data are the mean ± SEM. *P < 0.05, **P < 0.01, ***P < 0.001 by two-way ANOVA and Bonferroni *post hoc*.

Our previous investigations reported that treatment with epothilone or our rehabilitation paradigm does not affect lesion size or tissue sparing following mild contusive SCI.^24,25,30^ We therefore initially tested their effects in a more severe contusion injury model. Following a 175 kdyn contusion injury at T10, rats received either epoD (1.50 mg/kg), epoB (0.75 mg/kg) or vehicle (1:1 DMSO:saline) on Days 1 and 15 post-injury (Fig. 2A and B). A growth-promoting approach in parallel with rehabilitation can have counterproductive effects on functional recovery, whereas a sequential treatment showed an improvement.^37^ Hence, we started rehabilitation training with the animals of the rehabilitation group 3 weeks post-injury for 7 weeks. All animals in the study were perfused after 10 weeks prior to cryosectioning for IHC analysis (Fig. 2A and B). No differences in lesion size or spared tissue determined from the demarcation of GFAP immunostaining were observed between the groups (Fig. 2C–E). At the lesion centre, we observed a maximal lesion size of ∼1.0 mm^2^, surrounded by an area of spared tissue of ∼1.5 mm^2^.

Epothilones protect against dopaminergic neuron loss in rodent models of Parkinson’s disease.^38,39^ However, when we tested whether epoD and epoB could protect spinal cord neuron loss within spared tissue, immunostaining for the neuronal marker NeuN showed a similar loss of neurons towards the lesion centre in all groups (Fig. 2F and G). Thus, neither epothilones nor rehabilitation affects lesion size or neuroprotection after SCI.

### Epothilone B reduces fibrotic scar components after moderate contusive injury

**Figure 3:**
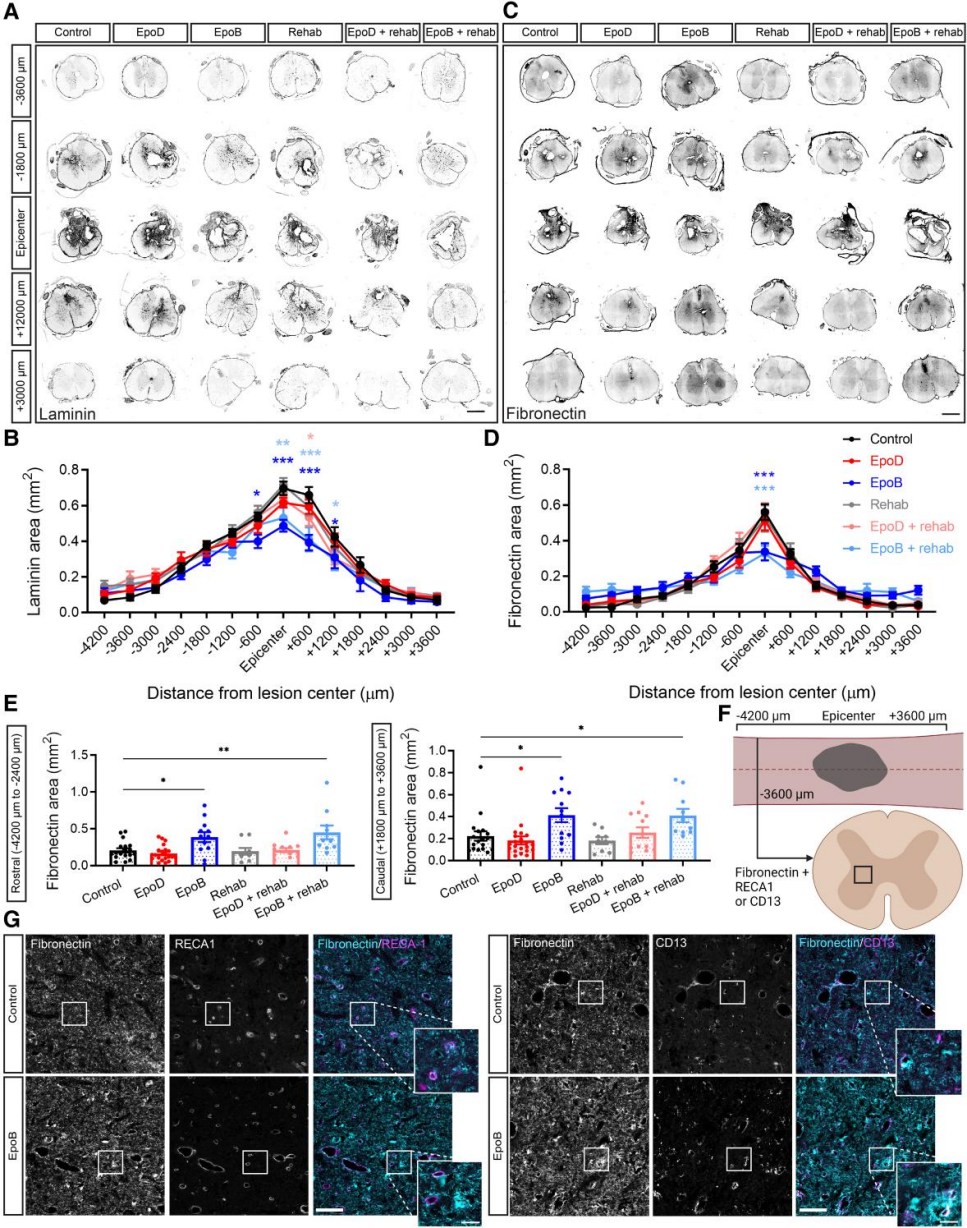
**Epothilone B reduces fibrotic scar components after T10 175 kdyn contusion injury**. (**A**) Representative fluorescent images of laminin throughout the lesioned spinal cord for the given groups (pixel values inverted; scale bar = 1 mm). (**B**) Quantification of laminin staining area within every 600 µm regions of spinal cord tissue (control *n* = 16; epoD *n* = 18; epoB *n* = 12; rehabilitation *n* = 11; epoD + rehabilitation *n* = 12; epoB + rehabilitation *n* = 11). (**C**) Representative fluorescent images of fibronectin throughout the lesioned spinal cord for the given groups (pixel values inverted; scale bar = 1 mm). (**D**) Quantification of fibronectin staining area within every 600 µm regions of spinal cord tissue. (**E**) Area of fibronectin staining in regions rostral (−4200 to −2400 µm) and caudal (+1800 to +3600 µm) to the lesion (Control *n* = 18; epoD *n* = 21; epoB *n* = 12; rehabilitation *n* = 9; epoD + rehabilitation *n* = 12; epoB + rehabilitation *n* = 10). (**F**) Schematic representation of the region in which IHC was performed for the colocalization of fibronectin to RECA1 and CD13. Created with BioRender.com. (**G**) Fluorescent images of spinal cord sections 3600 µm rostral from the lesion epicentre labelled for fibronectin and RECA-1 or CD13 (scale bar = 50 µm; 10 µm in zoomed panels). Plotted data are the mean ± SEM; each data point represents the mean per animal. *P < 0.05, **P < 0.01, ***P < 0.001 by two-way ANOVA and Bonferroni *post hoc.*

The fibrotic scar acts as a barrier to regenerating axons and its reduction is associated with behavioural improvements.^40^ Previous studies reported that epoB and epoD reduce fibrotic scarring after mild contusion SCI, when assessed 3 days and 4 weeks after injury.^24–26^ We, therefore, analysed fibrotic scarring in our more severe SCI model. We found that the fibrotic scar, as marked by laminin and fibronectin,^40^ was reduced after epoB treatment even 10 weeks after injury. Specifically, laminin immunoreactivity was reduced from 600 µm rostral to 1200 µm caudal to the lesion epicentre (Fig. 3A and B; *P* < 0.05; two-way ANOVA, Dunnett’s *post hoc*). For animals treated with epoD, only for those in position 600 µm caudal was this significant in the epoD + rehabilitation group (0.53 versus 0.65 mm^2^; *P* < 0.05). Animals that underwent rehabilitation alone showed no change in laminin immunoreactivity (Fig. 3A and B).

Consistently, fibronectin staining was also reduced at the lesion centre of epoB-treated animals (Fig. 3C and D; 0.34 versus 0.55 mm^2^; *P* < 0.0001; two-way ANOVA, Dunnett’s *post hoc*). Interestingly, we found an increase in fibronectin staining distal to the lesion in these epoB-treated animals (Fig. 3C). As EpoB reduces the migration of fibronectin-producing perivascular cells and promotes microcirculation reconstruction following SCI,^41^ we hypothesized that this increase in fibronectin could originate from cells localized to blood vessels, which expressed fibronectin but failed to migrate. EpoB-treated animals displayed greater fibronectin immunoreactivity in sections outside the lesion core in regions both rostral and caudal to the lesion compared with control animals (Fig. 3E; *P* < 0.05; see Supplementary Fig. 2 for the full data set).

It has been proposed that type A pericytes are largely responsible for fibronectin production after non-penetrating SCI.^42^ Consistent with this, IHC analysis rostral to the lesion using the endothelial cell marker rat endothelial cell antigen 1 (RECA-1) and the type A pericyte marker cluster of differentiation 13 (CD13) (Fig. 3F) showed that, compared with injured controls, epoB-treated animals displayed increased fibronectin immunoreactivity. This was largely distributed throughout the extracellular matrix and occasionally adjacent to vessels (Fig. 3G). Consistent with previous reports after epoB treatment,^41^ there appeared to be a greater number of RECA1-positive vessels and pericyte (CD13) counts which occasionally localized with fibronectin (Fig. 3G). Taken together, epoB has a persistent effect on decreasing fibrotic scarring at the lesion site and beyond in chronic SCI that may involve the prevention of perivascular cell migration and microvasculature reconstruction.

### Epothilone B reduces intralesional cell number

**Figure 4:**
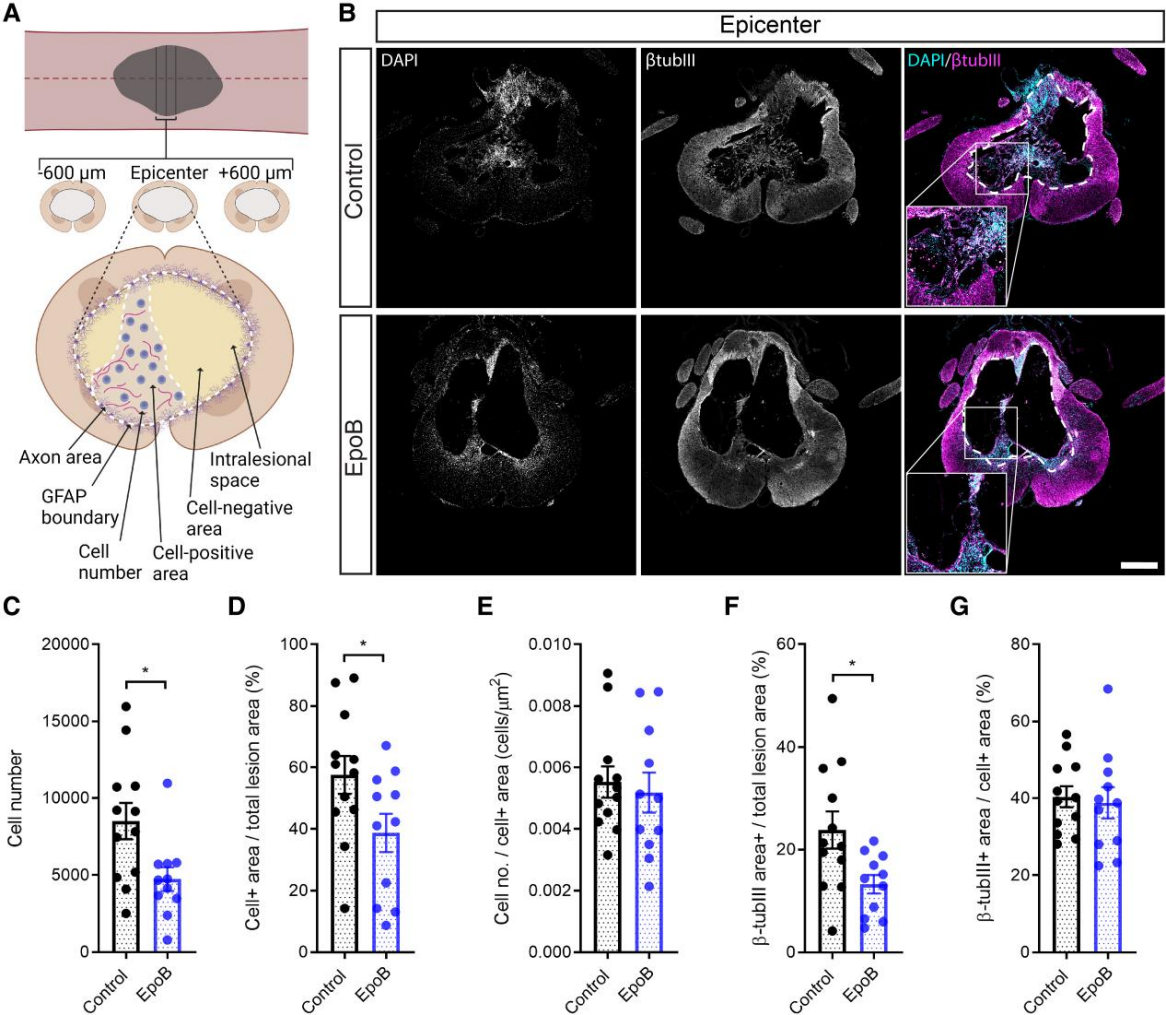
**Epothilone B reduces cell-positive lesion area and neuron expression within the lesion site**. (**A**) Schematic of the sections used and the parameters measured for the analysis. Created with BioRender.com. (**B**) Immunostaining for DAPI and βtubIII at the lesion epicentre in control animals or those treated with epoB (scale bar = 500 µm). (**C**) Quantification of the number of cells within the lesion. (**D**) Quantification of the percentage of cell-positive area to total lesion area. (**E**) Quantification of the percentage of βtubIII staining area to the total lesion area. (**F**) Quantification of the number of cells within cell-positive lesion area. (**G**) Quantification of the βtubIII staining area within cell-positive lesion area. Plotted data are the mean ±SEM; each data point represents the mean per animal. *P < 0.05 by Student’s *t*-tests. Control *n* = 12; epoB *n* = 11.

Considering epoB reduces the migration of pericytes and fibroblasts and potentially other cell types,^24,41^ we investigated whether there was a reduction of cell number within the lesion. Furthermore, as epoB reduces the presence of the fibrotic scar we also were interested in investigating what effect this had on axon regeneration within the lesion. To test this, we first delineated the total lesion area marked by the astrocytic boundary within sections taken at the lesion epicentre and 600 µm rostral and caudal from the lesion epicentre (Fig. 4A). Within the intralesional space, cell-positive and cell-negative (cavitation) areas were determined based on the cell nuclei (Fig. 4A and B). Albeit no change in the lesion size (Fig. 2), analysis of DAPI-positive cells showed that in animals treated with epoB, the intralesional space contained only half the cell numbers compared with those of control animals (Fig. 4C; *P* < 0.05; two-tailed *t*-test). Concomitantly, the ratio of cell-positive area to the total lesion area was reduced in epoB-treated animals by one-third (Fig. 4D; 38.7% versus 57.6%; *P* < 0.05), while the number of cells within the cell-positive lesion area remained unchanged (Fig. 4E). Consequently, axons marked by βtubIII within the total lesion area were reduced in the epoB-treated group (Fig. 4F; 13.25 versus 23.80%; *P* < 0.05). This was independent of the presence of axons within the cell-positive area (Fig. 4G): there, the axon area remained unchanged. Thus, epoB decreases cell number within the intralesional space and increases the proportion of cystic cavities to tissue; in cell-positive areas, cell number and axons remained unchanged.

### Epothilone B increases serotonergic fibre density caudal to the lesion site

**Figure 5:**
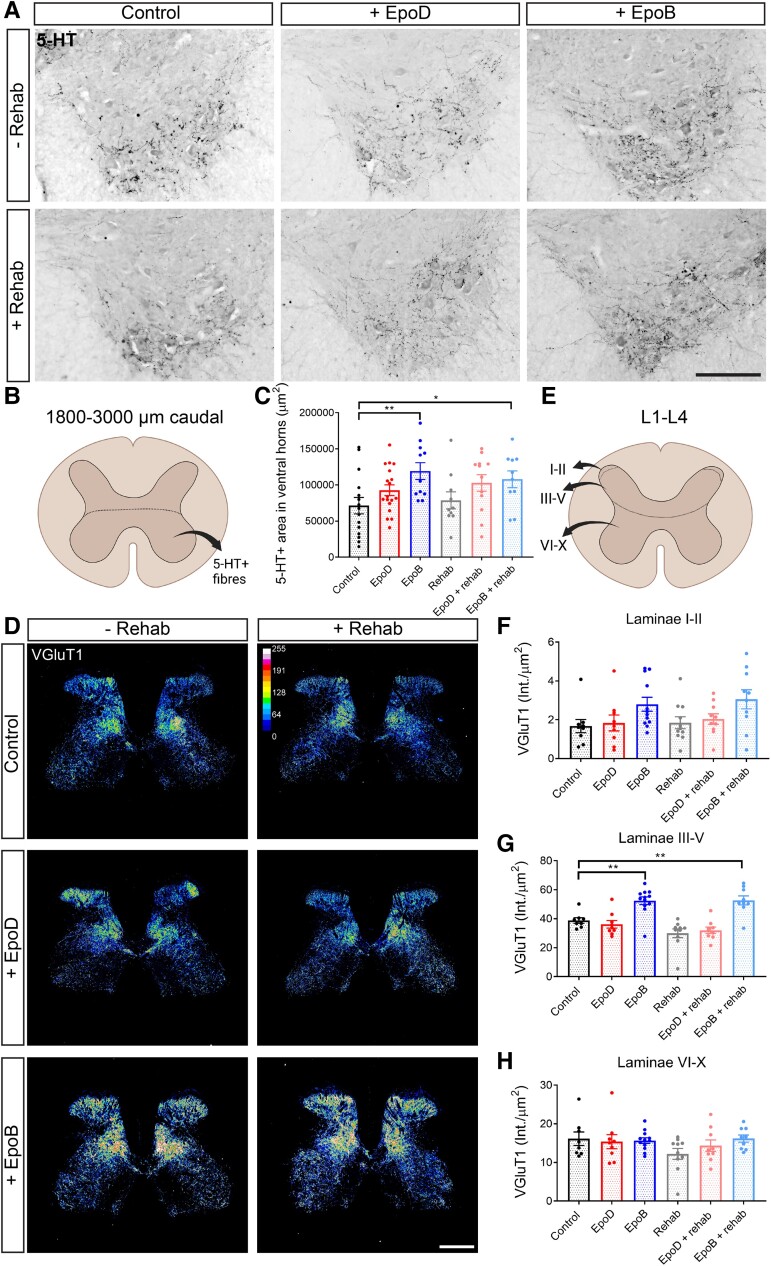
**Epothilones but not rehabilitation promotes serotonergic and lumbar VGluT1 plasticity**. (**A**) Representative images of sections 1800–3000 µm caudal to the lesion immunolabelled for 5-HT expression (pixel values inverted; scale bar = 200 µm). (**B**) Scheme depicting the ventral horn region in which images were thresholded to positive staining area and total 5-HT + immunolabelling within the ventral horns were measured. Created with BioRender.com. (**C**) Quantification of 5-HT staining area in the ventral horns in the given treatment groups (control *n* = 15; epoD *n* = 18; epoB *n* = 11; rehabilitation *n* = 10; epoD + rehabilitation *n* = 12; epoB + rehabilitation *n* = 10). (**D**) Representative fluorescent images of spinal cord sections immunolabelled for VGluT1 (grey value intensity gradient; scale bar = 500 µm) in the given treatment groups in coronal sections from lumbar spinal cord (L1–L4). (**E**) Scheme depicting that the quantification for VGluT1 was segregated into Laminae I–II, III–V and V–IX. Created with BioRender.com. (**F**–**H**) Quantification of VGluT1 immunolabelling per area in the various laminae in the given treatment groups (control *n* = 9; epoD *n* = 9; epoB *n* = 12; rehabilitation *n* = 11; epoD + rehabilitation *n* = 10; epoB + rehabilitation *n* = 10). Plotted data are the mean ±SEM; each data point represents the mean per animal. *P < 0.05, **P < 0.01, ***P < 0.001 by repeated-measures two-way ANOVA Dunnett’s and Bonferroni *post hoc.*

Five-HT projections descend from the raphe nuclei and terminate in the ventral horn of the spinal cord to provide neuromodulatory input to spinal interneurons and motor neurons. The loss of these projections correlates with locomotor dysfunction, and effective treatments for SCI often result in increased 5-HT fibre density caudal to the lesion.^43,44^ Through promoting axon growth, epoB and epoD induce this effect after hemisection or mild contusion injury.^24,25^ Likewise, some studies report serotonergic fibre sprouting after rehabilitation,^45,46^ although this was not observed in our previous study nor other studies.^14,30,47^ Therefore, we compared the two epothilones in our 175 kdyn contusion model and tested whether there was a further effect on serotonergic plasticity when given in combination with rehabilitation. Specifically, we performed 5-HT IHC on coronal sections 1800–3000 μm caudal to the lesion site, representing sections at approximately spinal cord levels T12–T13 (Fig. 5A), and measured the area of 5-HT + fibres within the ventral horns of these sections (Fig. 5B and C).

EpoB treatment alone resulted in approximately twice the amount of 5-HT-positive fibres compared to control animals (Fig. 5A and C; *P* < 0.05; one-way ANOVA, Dunnett’s *post hoc*). We found a comparable increase in 5-HT expression when epoB treatment was combined with rehabilitation. In contrast, consistent with our previous investigation,^30^ rehabilitation alone did not affect the 5-HT fibre density (*P* = 0.858; one-way ANOVA, Dunnett’s *post hoc*). EpoD treatment—both alone and in combination with rehabilitation—showed a trend towards an increased amount of 5-HT + fibres compared with the control animals (Fig. 5A and C; 92 705 versus 65 510 μm^2^; *P* = 0.1553; 102 732 versus 65 510 μm^2^; *P* = 0.0508; one-way ANOVA, Dunnett’s *post hoc*). Thus, epoB alone or epoB in combination with rehabilitation increased serotonergic axons after a moderate contusion injury.

### Epothilone B increases medial grey matter vesicular glutamate transporter 1 expression in the lumbar spinal cord

Rehabilitation has been proposed to improve functional recovery via a variety of mechanisms, including muscle mass and strength increase, BDNF (Brain Derived Neurotrophic Factor) upregulation, enhanced axon regeneration and synaptic plasticity promotion.^28^ SCI disrupts excitatory synaptic input responsible for locomotion. Recent reports show that rehabilitation in the form of epidural stimulation, along with daily step-training, results in excitatory synaptic remodelling of motor neurons, as measured by an increase in VGluT2 boutons following complete T9–T10 transection injury.^48^ Additionally, promoting plasticity through CSPG degradation in the injured spinal cord increases VGluT1 expression rostral and caudal to SCI.^49^ Therefore, we asked whether rehabilitation may exert an effect at the level of excitatory synapses that would lead to enhanced functional recovery and investigated the expression of presynaptic excitatory VGluT1 in the lumbar region. As the expression pattern of VGluT1 is strong within Laminae III–V and weak in Laminae I–II and VI–X, we segregated our analysis into these three regions (Fig. 5D–H). We found that rehabilitation did not affect VGluT1 expression nor did epoD treatment (Fig. 5F–H). Intriguingly, however, we found that in Laminae levels III–V, epoB-treated animals—alone or in combination with rehabilitation—had increased VGluT1 immunoreactivity compared with control animals (Fig. 5G; *P* = 0.0031: one-way ANOVA, Dunnett’s *post hoc*). The staining pattern of these animals is concentrated in Lamina III and medially in Laminae IV–V (Fig. 5D). Neither epoD nor rehabilitation influenced the expression of VGluT1 staining (Fig. 5F–H). Taken together, epoB alone or in combination with rehabilitation showed an increase in excitatory VGluT1 expression in medial grey matter.

### Functional recovery of spinal cord–injured rats after rehabilitation and epothilone B treatment

We then investigated functional recovery of the contused animals. To this end, we tested the rats for BBB locomotion and horizontal ladder walk. Then we utilized MotoRater kinematic profiling of the hindlimbs and Catwalk XT gait analysis, followed by multiparametric analyses to comprehensively analyse hindlimb kinematics and parameters pertaining to gait.

#### Rehabilitation improves open-field locomotion and ladder walking

**Figure 6:**
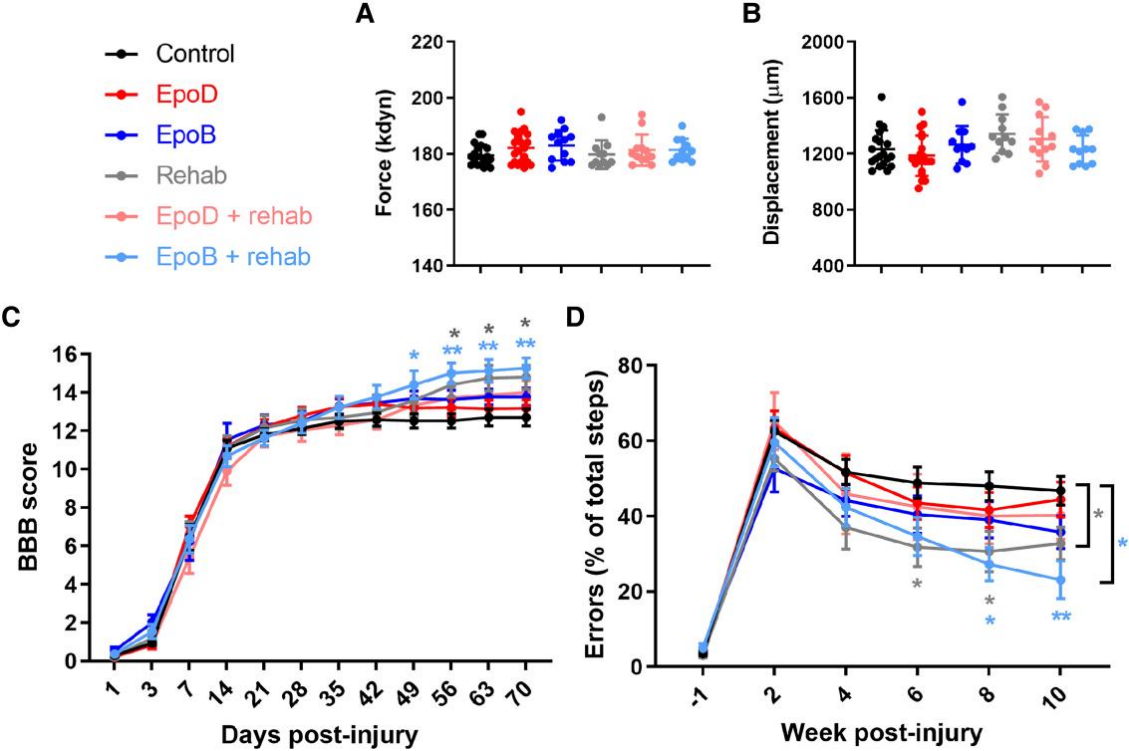
**Rehabilitation and a combination of rehabilitation with epothilone B promote recovery in open field and ladder tasks**. (**A** and **B**) The actual force and displacement of each impaction were plotted by one-way ANOVA, Bonferroni *post hoc*. (**C** and **D**) BBB and horizontal error ladder scores measured throughout the study. Plotted data are the mean ±SEM; each data point represents the mean per animal. *P < 0.05, **P < 0.01 by repeated-measures two-way ANOVA Dunnett’s and Bonferroni *post hoc* (light blue asterisks compare control with epoB = rehab; grey asterisks compare control with rehab). Control *n* = 17; epoD *n* = 20; epoB *n* = 11; rehabilitation *n* = 10; epoD + rehabilitation *n* = 12; epoB + rehabilitation *n* = 11.

Rats were scored for BBB locomotion and on the horizontal error ladder for 10 weeks post-injury. Measurements of force and displacement from the contusion impaction device confirmed injuries were of equal severity across treatment groups (Fig. 6A and B; *P* > 0.05; one-way ANOVA). All animals displayed a BBB score of less than two 1 day after injury, representing a state of complete paralysis to very limited movements (Fig. 6C). Animals spontaneously recovered hindlimb function over the following weeks; by week 2, animals performed weight-supported stepping, which is essential for the ladder task (BBB score of 10 or greater). Whilst epothilone-treated animals trended towards higher mean BBB scores compared to the control group, there was no significant improvement (Fig. 6C). In contrast, animals that received rehabilitation displayed significantly improved BBB scores from week 8 onwards (14.4 versus 12.5; *P* < 0.05; repeated measures two-way ANOVA, Dunnett’s *post hoc*). By week 10, animals that received rehabilitation improved by two BBB points, indicating animals regained coordinated stepping and parallel paw position at initial contact, compared to occasional coordinated stepping observed in control animals (14.8 versus 12.7; *P* = 0.0164: respeated measures two-way ANOVA, Dunnett’s *post hoc*). Interestingly, animals that received both epoB and rehabilitation showed these improvements already from week 7 onwards (14.4 versus 12.5; *P* < 0.05; repeated measures two-way ANOVA, Dunnett’s *post hoc*) and at week 10 the mean BBB score of 15.27 was higher than in all other experimental groups.

We next assessed hindlimb function using the horizontal ladder test with irregular gaps, a skilled motor task utilizing coordination, proprioception and fine paw function. Prior to the injury, <5% of all steps resulted in errors for all animals (Fig. 6D). Two weeks after injury, animals made an error in 50–70% of their steps, with the number of errors decreasing to varying degrees in a group-dependent manner over the subsequent 8 weeks. Animals that received rehabilitation alone made fewer errors in weeks 6 and 8 post-injury compared with the control animals (31.7 versus 48.9%, *P* = 0.0465; 30.6 versus 48.0%, *P* = 0.0414; repeated measures two-way ANOVA, Dunnett’s *post hoc*). Comparably, animals that received rehabilitation combined with epoB treatment made significantly fewer errors in weeks 8 and 10 post-injury compared with the control animals (27.3 versus 48.0%, *P* = 0.0112; 23.1 versus 46.8%, *P* = 0.0024; repeated measures two-way ANOVA, Dunnett’s *post hoc*), although this was not significantly different from the rehabilitation alone group. Therefore, in these two behavioural measures, rehabilitation improves performance.

#### Epothilone B treatment alters hindlimb kinematic profiles

**Figure 7:**
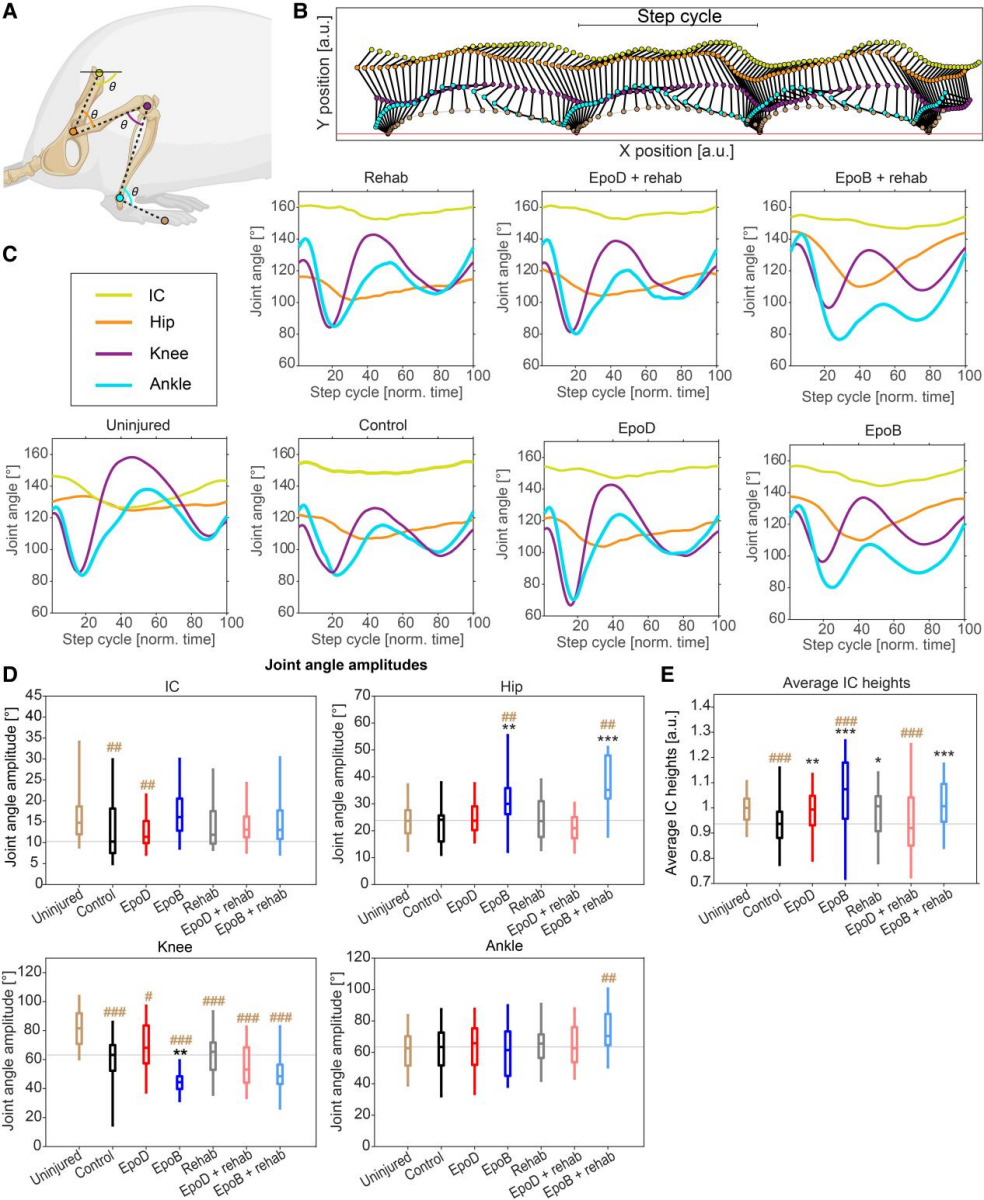
**Epothilone B improves aspects of hindlimb kinematics**. (**A**) Schematic of the five measured marker positions on the hindlimbs. For each video frame, these marker positions were used to calculate joint angles as depicted here. Created with BioRender.com. (**B**) An example of a complete walking trial for a right hind leg (uninjured condition). Marker colours correspond to those in (**A**). Complete step cycles have been defined as the movement from a lift-off event to the next. (**C**) Time course of all joint angles [see (**A**)] averaged over all steps for each individual and then averaged for all treatment groups separately (only left body side shown), normalized to their respective step cycles (see Supplementary Fig. 5 for averages of all individuals and both body sides). (**D**) Absolute amplitudes (difference between the maximum and minimum value during a step cycle) of all individual joint angles in all conditions (*P < 0.05, **P < 0.01, ***P < 0.001, Wilcoxon rank-sum test; black asterisks compare with control whereas brown asterisks compare with uninjured). (**E**) Average height of the IC marker above the surface of the walkway in all individual trials. Height was normalized to the height in the baseline condition. Boxplots indicate median, 25th, and 75th percentile (box) and 5th and 95th percentile (whiskers). Uninjured = 31; control *n* = 9; epoD *n* = 10; epoB *n* = 11; rehabilitation *n* = 10; epoD + rehabilitation *n* = 12; epoB + rehabilitation *n* = 11.

We assessed hindlimb kinematics during step cycles by tracking the marked joints: IC, hip, knee, ankle and toe (Fig. 7A). Each trial was segmented into a series of individual step cycles of the left and right hind leg, separately (Fig. 7B). We calculated the four joint angles (Fig. 7A), normalized each step to unit time, and then averaged all steps of the two hind legs in each animal to arrive at an average joint angle time courses for each leg (Fig. 7C; see Supplementary Fig. 3 for the complete data set). Uninjured animals showed stereotypical joint angle time courses. Specifically, the IC and hip joints displayed small, mono-phasic modulation over the step cycle, while knee and ankle presented greater amplitudes of movement, which were modulated in a bi-phasic manner (Fig. 7C and D). In contrast, the injured groups showed a shift in values for IC and hip joint angles. Specifically, the average hip joint angle was lower, while the IC joint angle was higher (Fig. 7C–E). This was observed together with a general decrease in knee and IC angle amplitudes features consistent with a more crouched posture and a decreased range of hindlimb motion (Supplementary Video 1 and Fig. 4).

While no discernible effect in the joint time courses was observed after epoD or rehabilitation, animals treated with epoB displayed greater angles of the hip joint at the beginning and the end of the step cycle, and full amplitudes of the hip (Fig. 7C and D). Concomitantly, we observed a decreased amplitude of the knee angle (Fig. 7C and D; *P* < 0.01; Wilcoxon rank-sum test); together with an increased IC height (Fig. 7E; *P* < 0.001), this indicated a higher upright stance during locomotion, combined with decreased knee range of motion. Hence, this walking pattern may represent an adaptive mechanism for locomotor recovery in epoB-treated animals.

#### Rehabilitation and epothilone B improve gait parameters and are additive in combination

**Figure 8:**
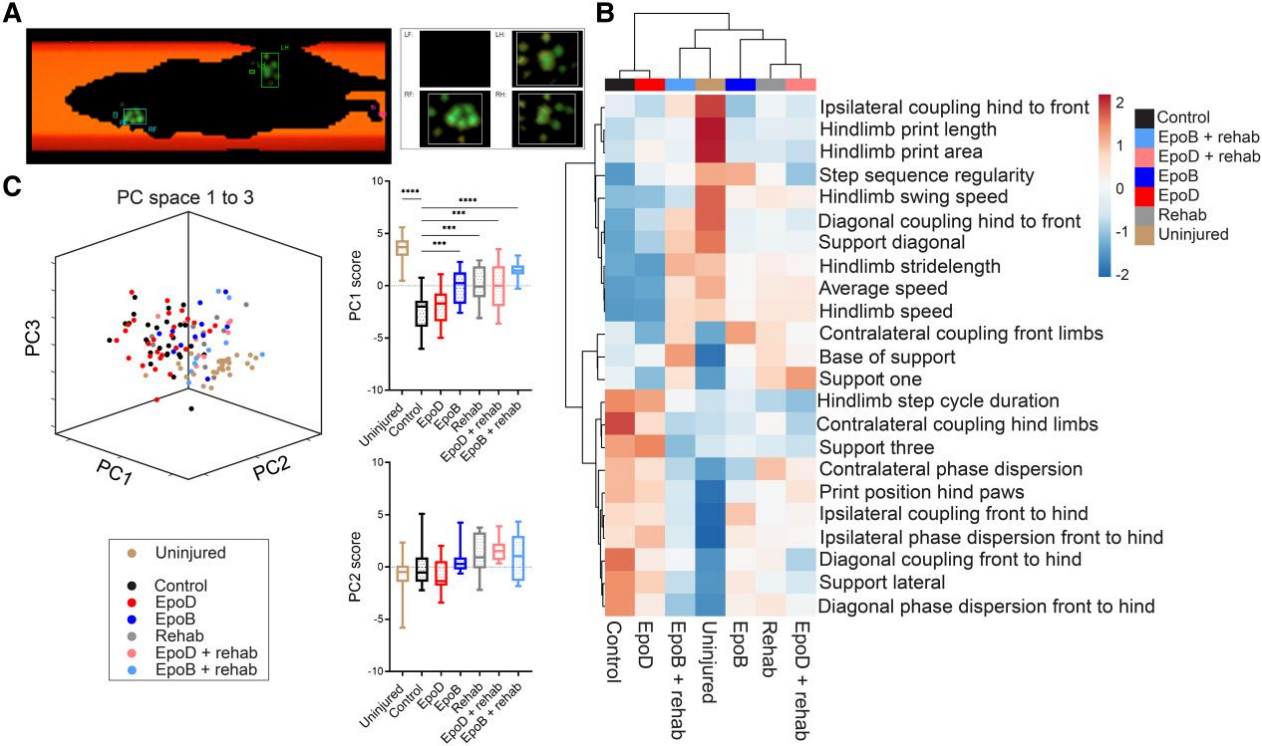
**The combination of rehabilitation with epothilone B restores parameters of gait through complementary locomotor mechanisms**. (**A**) An example image of the Catwalk XT gait analysis system recording paw prints during a run. (**B**) Heatmap hierarchical clustering of the PCA data set for correlation of parameters between treatment groups. (**C**) Three-dimensional depiction of the projection of all individual trials (data points represents means per animal) to the first three PCs and scores for PCs 1 and 2 (see also Supplementary Fig. 7). Boxplots indicate the median, 25th and 75th percentile (box) and 5th and 95th percentile (whiskers). *P < 0.05, **P < 0.01, ***P < 0.001, ****P < 0.0001 by repeated-measures two-way ANOVA Dunnett’s *post hoc*. Uninjured = 31; control *n* = 28; epoD *n* = 28; epoB *n* = 11; rehabilitation *n* = 10; epoD + rehabilitation *n* = 12; epoB + rehabilitation *n* = 10.

To better understand how gait was improved after the treatments, we finally performed catwalk gait analysis. Specifically, paw prints were detected by a high-speed camera, and print and gait parameters for the forelimbs and hindlimbs were digitally recorded (Fig. 8A). Parameters were assigned classifications. The data set was then pooled, becoming the basis for PCA and hierarchical heatmap clustering (Fig. 8B).

Compared with uninjured animals, several parameters were not improved in any treatment group, including hindlimb print area, print length, and ipsilateral coupling of the hindlimbs to the forelimbs (Fig. 8B; Supplementary Fig. 5). EpoD did not improve any parameters and, therefore, clustered to the control group in the heatmap cluster plot. In contrast, we found several parameters improved by epoB and by rehabilitation alone, including diagonal limb coupling and support, stride length, hindlimb speed, three-limb support and lateral limb support (Supplementary Fig. 5; *P* < 0.05; one-way ANOVA, Dunnett’s *post hoc*). Rehabilitation appeared to have a stronger effect on coordination and limb support scores, evident by this group solely improving one limb and lateral limb support as well as resulting in greater mean improvements to the majority of limb coupling scores (Fig. 8B; Supplementary Fig. 5).

Intriguingly, the combination of epoB and rehabilitation further improved gait parameters which is reflected by the close heatmap clustering of these two groups (Fig. 8B). Specifically, this combination resulted in the greatest improvement to the parameters diagonal coupling, diagonal support, stride length, hindlimb speed, three-limb support, print position, lateral support and diagonal phase dispersion (Fig. 8B; Supplementary Fig. 5; *P* < 0.01; one-way ANOVA, Dunnett’s *post hoc*).

We then extracted the PC scores for each treatment groups and determined the parameters that correlated within each PC. PC 1 alone described one-third of the variance of the data set (Supplementary Fig. 6A). Highly correlated parameters in PC1 included diagonal limb coupling, diagonal limb support and general locomotor parameters, including stride length and speed (Supplementary Fig. 6B). All treatment groups varied almost exclusively along PC1, indicating that it provides a general evaluation measure of how a particular treatment group compares to baseline (Supplementary Fig. 7). PCs 2 and 3, instead, captured systematic, treatment-unrelated, interindividual differences (PC2), or unspecific and unresolving variability that manifests after injury (PC3; Supplementary Fig. 7).

Expectedly, uninjured animals scored highest on PC1 when compared with all other groups (Fig. 8C; Supplementary Fig. 7). Conversely, the injured control animals scored lowest on PC1 (Fig. 8C; Supplementary Fig. 7). Improvements to PC1 scores were witnessed in the epoB, rehabilitation, epoD plus rehabilitation and the epoB plus rehabilitation (*P* < 0.0001) treatment groups (Fig. 8C; Supplementary Figure 7; *P* < 0.001 and *P* < 0.0001; one-way ANOVA, Dunnett’s *post hoc*). The greatest improvement, however, was to the epoB plus rehabilitation group compared with control animals which were most similar to the uninjured group. Thus, the parameters of diagonal limb coupling, diagonal limb support and general locomotor parameters, including stride length and speed, are holistically and additively improved in this combination group.

## Discussion

Previous work suggests that microtubule stabilization combined and rehabilitation may be both therapeutic avenues with translational potential for SCI.^24–26,30,50^ Here, we tested their combinatorial potential and found that systemic epoB administration followed by rehabilitation-enhanced functional recovery.

### The entry of epothilones into the CNS

EpoD and epoB completed human clinical trials when developed as anticancer agents; currently, ixabepilone is the only member of the epothilone family that is used in the clinic due to its particular efficacy in the context of metastatic breast cancer.^51^ Our data uncover that ixabepilone only weakly penetrates CNS compartments. Consistent with our finding, ixabepilone is a substrate for P-glycoprotein efflux pumps present on endothelial cells of the blood–brain barrier, a crucial mechanism for drug exclusion from the CNS.^52^ This contrasts the low susceptibility of epoB and epoD to P-glycoprotein-mediated drug exclusion.^51,52^ Thus, future investigations would need to test whether ixabepilone could have beneficial effects when applied intrathecally before it can be considered in future CNS repair therapies.

Instead, epoD and epoB are strongly BBB-permeable and rapidly distributed to the CNS compartments following i.p. or subcutaneous administration. However, they differed in their retention in the CNS: epoB persisted in the brain and spinal cord compartments for more than 2 weeks; in contrast, epoD was cleared from these compartments within 1 week. This PK difference in the epothilones is likely the reason for key differences observed in their actions. In this context, it is interesting to note that epoB has been successfully trialled in a Phase I/II study for recurrent glioblastoma.^53^

### The effect of epothilones on fibrotic scarring and intralesional cell number

Three days post-injury, perivascular cells proliferate, migrate and aggregate in the injured area where fibronectin and laminin are assembled at the lesion centre,^40^ a response attributed to perivascular type A pericytes.^42^ Previous work showed that the effect of epoB at reducing laminin is stronger 4 days post-injury compared with 1 month after an injury, consistent with the timing of cell migration to the lesion centre.^24^ Importantly, here, we observed a strong reduction of both laminin and fibronectin expression at the lesion centre by epoB, even 10 weeks following a more severe injury. Hence, the reduction of fibrotic scarring induced by epoB persists into the chronic stage of injury. Comparatively, the rapid clearance rate of epoD reduces the efficacy of the drug, preventing an ongoing process of extracellular matrix remodelling under the current dosing regimen. Hence, in the event that both epothilones would be followed up in studies towards an SCI therapy, our data suggest that epoD would have to be given at a more frequent dosing than epoB.

One way to interpret the reduction of fibrotic scarring is that epothilone-mediated microtubule stabilization prevents migration of cells involved in this process. Consistent with this possibility, we found in animals treated with epoB, extracellular and perivascular fibronectin staining in sections distal to the lesion and observed fewer cells in the intralesional space. Furthermore, following complete spinal cord transection in rats, epoB prevents the migration of pericytes and endothelial cells distal to the lesion site; this improved vascular regeneration.^41^ EpoB enables the proliferation and survival of pericytes and endothelial cells through upregulated expression of vascular endothelial growth factor A, vascular endothelial growth factor receptor 2, platelet-derived growth factor-β (PDGFβ), platelet-derived growth factor receptor β and angiopoietin-1.^41^ Recent lineage tracing experiments reported that the primary source of fibrotic scar-forming cells within the lesion are of type A pericyte origin.^42^ Thus, these data suggest the notion that pericytes are involved in fibrotic scarring and that epoB reduces their impact by preventing their migration.

Interventions that interfere with fibrotic scarring render tissue more permissive for axon regeneration and are associated with functional improvements.^54,55^ However, fibronectin and laminin are pro-regenerative and stimulate wound-healing effects.^56,57^ While our data highlight a reduction of axons in the intralesional space in the cell-positive area in this study, and as seen in previous studies,^24–26^ we also show that epoB promotes plasticity within tissue distal to the lesion. Thus, these data pinpoint the fact that it is still unclear whether the intralesional or the extralesional component of anatomical regeneration is involved in functional recovery. Future work is prerequisite to dissect the dichotomous roles of these fibrotic molecules in the context of SCI.

### The effect of epothilones and rehabilitation on spinal plasticity

Spinal cord serotonin levels facilitate locomotion and may influence functional recovery after SCI.^43^ EpoB increased serotonergic fibres caudal to the lesion site. Consistent with our previous investigation,^30^ rehabilitation alone did not affect the 5-HT fibre density. While this was also not observed in other studies investigating rehabilitation,^14,47^ there are reports of the contrary.^45,46^ The reason for this dichotomy may be the result of the varying forms in which rehabilitation can be applied or species differences between mice and rats. Intriguingly, we found that epoB also increased VGluT1 expression in Laminae III–V, a novel mechanism associated with microtubule stabilization. How microtubule stabilization induces this increase remains to be characterized. As previous reports showed that epoB decreases CSPG production and deposition^24^ and that CSPG degradation increases VGluT1 expression,^49^ thus it is possible that epoB increases VGluT1 expression through CSPG decrease. Alternatively, microtubule stabilization enables increased cargo transport in the axon and may thereby more efficiently deliver VGluT1.^58^

VGluT1-expressing primary afferent terminals in the superficial and deep dorsal horn are associated with proprioception and the transmission of tactile stimuli.^59^ In this context, it is noteworthy that proprioceptive feedback below the lesion drives spontaneous locomotor recovery.^60^ Therefore, epoB could influence recovery by enhancing proprioceptive signals driving activity-dependent connectivity changes. VGlut1/2-expressing interneurons in Laminae III–V also control and modulate motor neurons.^61^ As such, behavioural improvements after epoB treatment could be attributed to strengthening excitatory networks.

### Combining epothilone B and rehabilitation: enhancement of locomotor recovery

Epothilones improve outcomes on behavioural tasks following SCI and stroke models.^24–27^ Similarly, rehabilitative strategies improve function alone and can enhance functional recovery in combination with a regeneration-promoting therapy.^28^ Rehabilitation comes in many forms; our paradigm consisted of bipedal and quadrupedal training. This blend of training was selected to reinforce supraspinal-controlled initiation and modulation of bipedal locomotion,^62^ as well as the reorganization and re-engagement of rostrocaudal spinal interneuron networks following quadrupedal training.^63^ This helped us to uncover that epoB and rehabilitation improve both similar and complementary parameters of locomotion. Specifically, rehabilitation more strongly affected aspects of coordination and limb support scores; epoB treatment improved these parameters as well, albeit to a lesser extent, but uniquely altered the kinematic profile of hindlimb step cycles. When epoB and rehabilitation were combined, however, the PCA of gait showed the greatest improvement when compared with control animals, due to the overlapping benefits exerted from each intervention.

How might these therapies be interacting to produce these effects? Microtubule stabilization promotes axon regeneration/sprouting of several types of neurons *in vitro*—including cortical, hippocampal and cerebellar granular neurons—and *in vivo*—including dorsal root ganglion and raphe spinal neurons.^22–24,54^ We can therefore hypothesize that epoB enhances regenerative responses of axons that spontaneously occur following SCI, resulting in a comparatively broad effect on behaviour. On the other hand, rehabilitation appears to selectively affect the neural circuits involved in rehabilitative movements.^14^ Notably, rehabilitation can also promote the formation of nascent circuits only possible through rehabilitative activities.^62,64^ Quadrupedal walking can occur in the complete absence of supraspinal input using electrochemical modulation^65^; yet effective coordination of the limbs during walking occurs through spinal locomotor networks, the interplay between the circuits of the forelimbs and the supraspinal structures that regulate posture and volitional aspects of locomotion.^66^ Finer movements, including placement of paws and control of the digits or bipedal locomotion, largely requires higher level functioning or supraspinal connectivity to lumbosacral circuits.^62,67^ Taken together, rehabilitation could strengthen the neuronal circuitry specifically involved in bipedal and quadrupedal locomotion that has been promoted by the broad regenerative effects of epoB.

## Conclusions

Rehabilitation and epoB improve differing aspects of function and their combination can be further therapeutic. This combination strategy could therefore represent a promising avenue for future clinical translation.

## Supplementary Material

fcad005_Supplementary_Data

## Data Availability

Raw data were generated at The German Center for Neurodegenerative Diseases. Derived data supporting the findings of this study are available from the corresponding authors on request.

## Abbreviations

ARRIVE =: Reporting of *in vivo* experiments
5-HT =: serotonin
BBB =: blood–brain barrier
BBB score =: Basso, Beattie and Bresnahan locomotor scale score
BDNF =: brain derived neurotrophic factor
βtubIII =: β-tubulin-III
CSPG =: chondroitin sulphate proteoglycan
CMOS =: complementary metal-oxide semiconductor
DAPI =: 4′,6-diamidino-2-phenylindole
DMSO =: dimethyl sulfoxide
epoB =: epothilone B
epoD =: epothilone D
FDA =: Food and Drug Administration
GFAP =: glial fibrillary acidic protein
IC =: iliac crest
IHC =: immunohistochemistry
i.p. =: intraperitoneal
LC–MS =: liquid chromatography–mass spectrometry
NeuN =: neuronal nuclei
PBS =: phosphate buffered saline
PBST =: phosphate buffered saline with 0.2% triton X-100
PC =: principal component
PCA =: principal component analysis
PK =: pharmacokinetic
SCI =: spinal cord injury
s.c. =: subcutaneous
SEM =: standard error of the mean
UHPLC =: ultra high performance liquid chromatography
VGluT =: vesicular glutamate transporter

## References

[1] Curcio M , Bradke F. Axon regeneration in the central nervous system: Facing the challenges from the inside. Annu Rev Cell Dev Biol. 2018;34:495–521. 30044649 10.1146/annurev-cellbio-100617-062508

[2] Bradbury EJ , Burnside ER. Moving beyond the glial scar for spinal cord repair. Nat Commun. 2019;10(1):3879. 31462640 10.1038/s41467-019-11707-7 PMC6713740

[3] Silver J , Schwab ME, Popovich PG. Central nervous system regenerative failure: Role of oligodendrocytes, astrocytes, and microglia. Cold Spring Harb Perspect Biol. 2014;7(3):a020602. 10.1101/cshperspect.a020602 PMC4355267 25475091

[4] Rosenzweig ES , Salegio EA, Liang JJ, et al Chondroitinase improves anatomical and functional outcomes after primate spinal cord injury. Nat Neurosci. 2019;22(8):1269–1275. 31235933 10.1038/s41593-019-0424-1 PMC6693679

[5] Warren PM , Steiger SC, Dick TE, MacFarlane PM, Alilain WJ, Silver J. Rapid and robust restoration of breathing long after spinal cord injury. Nat Commun. 2018;9(1):4843. 30482901 10.1038/s41467-018-06937-0 PMC6258702

[6] Brommer B , He M, Zhang Z, et al Improving hindlimb locomotor function by non-invasive AAV-mediated manipulations of propriospinal neurons in mice with complete spinal cord injury. Nat Commun. 2021;12(1):781. 33536416 10.1038/s41467-021-20980-4 PMC7859413

[7] Beaud ML , Rouiller EM, Bloch J, Mir A, Schwab ME, Schmidlin E. Combined with anti-Nogo-A antibody treatment, BDNF did not compensate the extra deleterious motor effect caused by large size cervical cord hemisection in adult macaques. CNS Neurosci Ther. 2020;26(2):260–269. 31418518 10.1111/cns.13213 PMC6978268

[8] Rosenzweig ES , Brock JH, Lu P, et al Restorative effects of human neural stem cell grafts on the primate spinal cord. Nat Med. 2018;24(4):484–490. 29480894 10.1038/nm.4502 PMC5922761

[9] Anderson MA , O’Shea TM, Burda JE, et al Required growth facilitators propel axon regeneration across complete spinal cord injury. Nature. 2018;561(7723):396–400. 30158698 10.1038/s41586-018-0467-6 PMC6151128

[10] Jin D , Liu Y, Sun F, Wang X, Liu X, He Z. Restoration of skilled locomotion by sprouting corticospinal axons induced by co-deletion of PTEN and SOCS3. Nat Commun. 2015;6:8074. 26598325 10.1038/ncomms9074 PMC4662086

[11] Griffin JM , Bradke F. Therapeutic repair for spinal cord injury: Combinatory approaches to address a multifaceted problem. EMBO Mol Med. 2020;12(3):e11505. 10.15252/emmm.201911505 PMC7059014 32090481

[12] Kleitman N . Keeping promises: Translating basic research into new spinal cord injury therapies. J Spinal Cord Med. 2004;27(4):311–318. 15484661 10.1080/10790268.2004.11753768

[13] Fawcett JW , Curt A. Damage control in the nervous system: Rehabilitation in a plastic environment. Nat Med. 2009;15(7):735–736. 19584862 10.1038/nm0709-735

[14] Garcia-Alias G , Barkhuysen S, Buckle M, Fawcett JW. Chondroitinase ABC treatment opens a window of opportunity for task-specific rehabilitation. Nat Neurosci. 2009;12(9):1145–1151. 19668200 10.1038/nn.2377

[15] Shinozaki M , Iwanami A, Fujiyoshi K, et al Combined treatment with chondroitinase ABC and treadmill rehabilitation for chronic severe spinal cord injury in adult rats. Neurosci Res. 2016;113:37–47. 27497528 10.1016/j.neures.2016.07.005

[16] Wang D , Ichiyama RM, Zhao R, Andrews MR, Fawcett JW. Chondroitinase combined with rehabilitation promotes recovery of forelimb function in rats with chronic spinal cord injury. J Neurosci. 2011;31(25):9332–9344. 21697383 10.1523/JNEUROSCI.0983-11.2011 PMC6623473

[17] Duan R , Qu M, Yuan Y, et al Clinical benefit of rehabilitation training in spinal cord injury: A systematic review and meta-analysis. Spine (Phila Pa 1976). 2021;46(6):E398–E410. 33620185 10.1097/BRS.0000000000003789

[18] Blanquie O , Bradke F. Cytoskeleton dynamics in axon regeneration. Curr Opin Neurobiol. 2018;51:60–69. 29544200 10.1016/j.conb.2018.02.024

[19] Costa AR , Sousa MM. Non-muscle myosin II in axonal cell biology: From the growth cone to the axon initial segment. Cells. 2020;9(9):1-17. 10.3390/cells9091961 PMC7563147 32858875

[20] Stern S , Hilton BJ, Burnside ER, et al Rhoa drives actin compaction to restrict axon regeneration and astrocyte reactivity after CNS injury. Neuron. 2021;109(21):3436–3455.e9. 34508667 10.1016/j.neuron.2021.08.014

[21] Tedeschi A , Dupraz S, Curcio M, et al ADF/Cofilin-mediated actin turnover promotes axon regeneration in the adult CNS. Neuron. 2019;103(6):1073–1085.e6. 31400829 10.1016/j.neuron.2019.07.007 PMC6763392

[22] Erturk A , Hellal F, Enes J, Bradke F. Disorganized microtubules underlie the formation of retraction bulbs and the failure of axonal regeneration. J Neurosci. 2007;27(34):9169–9180. 17715353 10.1523/JNEUROSCI.0612-07.2007 PMC6672197

[23] Witte H , Neukirchen D, Bradke F. Microtubule stabilization specifies initial neuronal polarization. J Cell Biol. 2008;180(3):619–632. 18268107 10.1083/jcb.200707042 PMC2234250

[24] Ruschel J , Hellal F, Flynn KC, et al Axonal regeneration. Systemic administration of epothilone B promotes axon regeneration after spinal cord injury. Science. 2015;348(6232):347–352. 25765066 10.1126/science.aaa2958 PMC4445125

[25] Ruschel J , Bradke F. Systemic administration of epothilone D improves functional recovery of walking after rat spinal cord contusion injury. Exp Neurol. 2018;306:243–249. 29223322 10.1016/j.expneurol.2017.12.001

[26] Sandner B , Puttagunta R, Motsch M, et al Systemic epothilone D improves hindlimb function after spinal cord contusion injury in rats. Exp Neurol. 2018;306:250–259. 29408734 10.1016/j.expneurol.2018.01.018

[27] Kugler C , Thielscher C, Tambe BA, et al Epothilones improve axonal growth and motor outcomes after stroke in the adult mammalian CNS. Cell Rep Med. 2020;1(9):100159. 10.1016/j.xcrm.2020.100159 PMC7762779 33377130

[28] Loy K , Bareyre FM. Rehabilitation following spinal cord injury: How animal models can help our understanding of exercise-induced neuroplasticity. Neural Regen Res. 2019;14(3):405–412. 30539806 10.4103/1673-5374.245951 PMC6334617

[29] du Sert N P , Hurst V, Ahluwalia A, et al The ARRIVE guidelines 2.0: Updated guidelines for reporting animal research. BMJ Open Sci. 2020;4(1):e100115. 10.1136/bmjos-2020-100115 PMC7610906 34095516

[30] Griffin JM , Fackelmeier B, Clemett CA, et al Astrocyte-selective AAV-ADAMTS4 gene therapy combined with hindlimb rehabilitation promotes functional recovery after spinal cord injury. Exp Neurol. 2020;327:113232. 10.1016/j.expneurol.2020.113232 32044329

[31] Basso DM , Beattie MS, Bresnahan JC. A sensitive and reliable locomotor rating scale for open field testing in rats. J Neurotrauma. 1995;12(1):1–21. 7783230 10.1089/neu.1995.12.1

[32] Zorner B , Filli L, Starkey ML, et al Profiling locomotor recovery: Comprehensive quantification of impairments after CNS damage in rodents. Nat Methods. 2010;7(9):701–708. 20836253 10.1038/nmeth.1484

[33] Hamers FP , Koopmans GC, Joosten EA. CatWalk-assisted gait analysis in the assessment of spinal cord injury. J Neurotrauma. 2006;23(3–4):537–548. 16629635 10.1089/neu.2006.23.537

[34] Aceves M , Dietz VA, Dulin JN, Jeffery U, Jeffery ND. An analysis of variability in “CatWalk” locomotor measurements to aid experimental design and interpretation. eNeuro. 2020;7(4):1-9. 10.1523/ENEURO.0092-20.2020 PMC7458803 32647037

[35] Pharmacology CG . Part 2: Introduction to pharmacokinetics. J Nucl Med Technol. 2018;46(3):221–230. 29724803 10.2967/jnmt.117.199638

[36] Information NCfB . PubChem compound summary for CID 448013, Epothilone B. Accessed 9 August 2022. https://pubchem.ncbi.nlm.nih.gov/compound/Epothilone-B.

[37] Wahl AS , Omlor W, Rubio JC, et al Neuronal repair. Asynchronous therapy restores motor control by rewiring of the rat corticospinal tract after stroke. Science. 2014;344(6189):1250–1255. 24926013 10.1126/science.1253050

[38] Cartelli D , Casagrande F, Busceti CL, et al Microtubule alterations occur early in experimental parkinsonism and the microtubule stabilizer epothilone D is neuroprotective. Sci Rep. 2013;3:1837. 23670541 10.1038/srep01837 PMC3653217

[39] Yu Z , Yang L, Yang Y, et al Epothilone B benefits nigral dopaminergic neurons by attenuating microglia activation in the 6-hydroxydopamine lesion mouse model of Parkinson’s disease. Front Cell Neurosci. 2018;12:324. 30323743 10.3389/fncel.2018.00324 PMC6172330

[40] Li Z , Yu S, Hu X, et al Fibrotic scar after spinal cord injury: Crosstalk with other cells, cellular origin, function, and mechanism. Front Cell Neurosci. 2021;15:720938. 10.3389/fncel.2021.720938 PMC8441597 34539350

[41] Duan YY , Chai Y, Zhang NL, Zhao DM, Yang C. Microtubule stabilization promotes microcirculation reconstruction after spinal cord injury. J Mol Neurosci. 2021;71(3):583–595. 32901373 10.1007/s12031-020-01679-5 PMC7851021

[42] Dias DO , Kalkitsas J, Kelahmetoglu Y, et al Pericyte-derived fibrotic scarring is conserved across diverse central nervous system lesions. Nat Commun. 2021;12(1):5501. 34535655 10.1038/s41467-021-25585-5 PMC8448846

[43] Schmidt BJ , Jordan LM. The role of serotonin in reflex modulation and locomotor rhythm production in the mammalian spinal cord. Brain Res Bull. 2000;53(5):689–710. 11165804 10.1016/s0361-9230(00)00402-0

[44] Perrin FE , Noristani HN. Serotonergic mechanisms in spinal cord injury. Exp Neurol. 2019;318:174–191. 31085200 10.1016/j.expneurol.2019.05.007

[45] Engesser-Cesar C , Ichiyama RM, Nefas AL, et al Wheel running following spinal cord injury improves locomotor recovery and stimulates serotonergic fiber growth. Eur J Neurosci. 2007;25(7):1931–1939. 17439482 10.1111/j.1460-9568.2007.05469.x

[46] Loy K , Schmalz A, Hoche T, et al Enhanced voluntary exercise improves functional recovery following spinal cord injury by impacting the local neuroglial injury response and supporting the rewiring of supraspinal circuits. J Neurotrauma. 2018;35(24):2904–2915. 29943672 10.1089/neu.2017.5544

[47] Maier IC , Ichiyama RM, Courtine G, et al Differential effects of anti-Nogo-A antibody treatment and treadmill training in rats with incomplete spinal cord injury. Brain. 2009;132(Pt 6):1426–1440. 19372269 10.1093/brain/awp085

[48] Al’joboori YD , Edgerton VR, Ichiyama RM. Effects of rehabilitation on perineural nets and synaptic plasticity following spinal cord transection. Brain Sci. 2020;10(11):1-13. 10.3390/brainsci10110824 PMC7694754 33172143

[49] Burnside ER , De Winter F, Didangelos A, et al Immune-evasive gene switch enables regulated delivery of chondroitinase after spinal cord injury. Brain. 2018;141(8):2362–2381. 29912283 10.1093/brain/awy158 PMC6061881

[50] Hilton BJ , Bradke F. Can injured adult CNS axons regenerate by recapitulating development? Development. 2017;144(19):3417–3429. 28974639 10.1242/dev.148312

[51] Cheng KL , Bradley T, Budman DR. Novel microtubule-targeting agents - the epothilones. Biologics. 2008;2(4):789–811. 19707459 10.2147/btt.s3487 PMC2727900

[52] Shen H , Lee FY, Gan J. Ixabepilone, a novel microtubule-targeting agent for breast cancer, is a substrate for P-glycoprotein (P-gp/MDR1/ABCB1) but not breast cancer resistance protein (BCRP/ABCG2). J Pharmacol Exp Ther. 2011;337(2):423–432. 21262849 10.1124/jpet.110.175604

[53] Oehler C , Frei K, Rushing EJ, et al Patupilone (epothilone B) for recurrent glioblastoma: Clinical outcome and translational analysis of a single-institution phase I/II trial. Oncology. 2012;83(1):1–9. 22688083 10.1159/000339152

[54] Hellal F , Hurtado A, Ruschel J, et al Microtubule stabilization reduces scarring and causes axon regeneration after spinal cord injury. Science. 2011;331(6019):928–931. 21273450 10.1126/science.1201148 PMC3330754

[55] Klapka N , Hermanns S, Straten G, et al Suppression of fibrous scarring in spinal cord injury of rat promotes long-distance regeneration of corticospinal tract axons, rescue of primary motoneurons in somatosensory cortex and significant functional recovery. Eur J Neurosci. 2005;22(12):3047–3058. 16367771 10.1111/j.1460-9568.2005.04495.x

[56] Li Y , He X, Kawaguchi R, et al Microglia-organized scar-free spinal cord repair in neonatal mice. Nature. 2020;587(7835):613–618. 33029008 10.1038/s41586-020-2795-6 PMC7704837

[57] Menezes K , de Menezes JR, Nascimento MA, Santos Rde S, Coelho-Sampaio T. Polylaminin, a polymeric form of laminin, promotes regeneration after spinal cord injury. FASEB J. 2010;24(11):4513–4522. 20643907 10.1096/fj.10-157628

[58] Seno T IT , Mennya K, Kurishita M, et al Kinesin-1 sorting in axons controls the differential retraction of arbor terminals. J Cell Sci. 2016;15(18):3499–3510. 10.1242/jcs.183806 27505885

[59] Brumovsky PR . VGLUTs in peripheral neurons and the spinal cord: Time for a review. ISRN Neurol. 2013;2013:829753. 10.1155/2013/829753 PMC3856137 24349795

[60] Takeoka A , Arber S. Functional local proprioceptive feedback circuits initiate and maintain locomotor recovery after spinal cord injury. Cell Rep. 2019;27(1):71–85.e3. 30943416 10.1016/j.celrep.2019.03.010

[61] Iizuka M , Ikeda K, Onimaru H, Izumizaki M. Expressions of VGLUT1/2 in the inspiratory interneurons and GAD65/67 in the inspiratory Renshaw cells in the neonatal rat upper thoracic spinal cord. IBRO Rep. 2018;5:24–32. 30135953 10.1016/j.ibror.2018.08.001 PMC6095097

[62] van den Brand R , Heutschi J, Barraud Q, et al Restoring voluntary control of locomotion after paralyzing spinal cord injury. Science. 2012;336(6085):1182–1185. 22654062 10.1126/science.1217416

[63] Shah PK , Garcia-Alias G, Choe J, et al Use of quadrupedal step training to re-engage spinal interneuronal networks and improve locomotor function after spinal cord injury. Brain. 2013;136(Pt 11):3362–3377. 24103912 10.1093/brain/awt265 PMC3808689

[64] Asboth L , Friedli L, Beauparlant J, et al Cortico-reticulo-spinal circuit reorganization enables functional recovery after severe spinal cord contusion. Nat Neurosci. 2018;21(4):576–588. 29556028 10.1038/s41593-018-0093-5

[65] Courtine G , Gerasimenko Y, van den Brand R, et al Transformation of nonfunctional spinal circuits into functional states after the loss of brain input. Nat Neurosci. 2009;12(10):1333–1342. 19767747 10.1038/nn.2401 PMC2828944

[66] Frigon A . The neural control of interlimb coordination during mammalian locomotion. J Neurophysiol. 2017;117(6):2224–2241. 28298308 10.1152/jn.00978.2016 PMC5454475

[67] Fouad K , Ng C, Basso DM. Behavioral testing in animal models of spinal cord injury. Exp Neurol. 2020;333:113410. 10.1016/j.expneurol.2020.113410 PMC8325780 32735871

