# Correction to: Rehabilitation enhances epothilone-induced locomotor recovery after spinal cord injury

**DOI:** 10.1093/braincomms/fcad029

**Published:** 2023-02-17

This is a correction to: Jarred M Griffin, Sonia Hingorani Jai Prakash, Till Bockemühl, Jessica M Benner, Barbara Schaffran, Victoria Moreno-Manzano, Ansgar Büschges, Frank Bradke, Rehabilitation enhances epothilone-induced locomotor recovery after spinal cord injury, *Brain Communications*, Volume 5, Issue 1, 2023, fcad005, https://doi.org/10.1093/braincomms/fcad005

In the originally published version of this manuscript online, the link to Video 1 was lacking in the supplementary data file. This lacuna has been supplied in the article online.

