# Supplementary material for: Rehabilitation enhances epothilone-induced locomotor recovery after spinal cord injury
Source: Brain Commun. 2023 Jan 13;5(1):fcad005. doi: 10.1093/braincomms/fcad005 (PMC9893225; doi:10.1093/braincomms/fcad005)
Supplement: fcad005_Supplementary_Data [file fcad005_supplementary_data.zip › Supplementary_Material.pdf]

Supplementary material

Rehabilitation enhances epothilone-induced locomotor recovery after spinal cord injury

Jarred M. Griffin, Sonia Hingorani Jai Prakash, Till Bockemühl, Jessica M. Gonyer, Barbara Schaffran, Victoria Moreno Manzano, Ansgar Büschges, and Frank Bradke.

Supplementary Table 1. List of antibodies used for immunohistochemistry

Antibody	Host	Company	Catalogue No.	Dilution
Anti-βtubulin III	Rabbit	Sigma	T2200	1:1000
Anti-CD13	Goat	R&D Systems	AF2335	1:1000
Anti-fibronectin	Rabbit	Abcam	AB1954	1:1000
Anti-GFAP	Mouse	Sigma	G3893	1:500
Anti-GFAP	Rabbit	DAKO	20334	1:1000
Anti-NeuN	Mouse	Merck Millipore	MAB377	1:500
Anti-laminin	Rabbit	Sigma	L9393	1:1000
Anti-RECA-1	Mouse	Sigma	MA1-81510	1:200
Anti-VGluT1	Rabbit	Synaptic Systems	135303	1:2000
Anti-VGluT2	Rabbit	Synaptic Systems	135403	1:2000
Anti-5HT	Rabbit	Sigma	S5545	1:1000
Anti-Mouse A488	Goat	ThermoFisher	A11029	1:500
Anti-Rabbit AF488	Donkey	ThermoFisher	A21206	1:500
Anti-Rabbit AF555	Goat	ThermoFisher	A21429	1:500
Anti-Rabbit AF594	Goat	ThermoFisher	A11037	1:500
Anti-Rabbit AF594	Donkey	ThermoFisher	A21207	1:500

Supplementary Table 2. List of parameters analysed in the Catwalk Gait analysis and their classifications

Parameters	Classification	Sub Classification
**Couplings RF LH**	Step Sequence	Diagonal Coupling
**Couplings LF RH**	Step Sequence	Diagonal Coupling
**Couplings RF RH**	Step Sequence	Ipsilateral Coupling
**Couplings LF LH**	Step Sequence	Ipsilateral Coupling
**Couplings RH LF**	Step Sequence	Diagonal Coupling
**Couplings LH RF**	Step Sequence	Diagonal Coupling
**Couplings LH RH**	Step Sequence	Inter-limb Coupling

**Step sequence regularity**	Step Sequence	Sequence
**Base of Support**	Support	BOS
**Support Three**	Support	Support Formula
**Support Lateral**	Support	Support Formula
**Support Diagonal**	Support	Support Formula
**Support One**	Support	Support Formula
**Print Position Right**	Paw Statistics	Print Position
**Print Position Left**	Paw Statistics	Print Position
**Right HL Stride length**	Paw Statistics	Stride Length
**Left HL Stride length**	Paw Statistics	Stride Length
**RH Print area**	Paw Statistics	Area
**LH Print area**	Paw Statistics	Area
**RH Swing speed**	Paw Statistics	Swing
**LH Swing speed**	Paw Statistics	Swing
**RH Step Cycle Duration**	General	Step Cycle
**LH Step Cycle Duration**	General	Step Cycle
**RH Print Width**	Paw Statistics	Width
**LH Print Width**	Paw Statistics	Width
**RH Stand**	Paw Statistics	Stand
**LH Stand**	Paw Statistics	Stand
**RH Print Length**	Paw Statistics	Length
**LH Print Length**	Paw Statistics	Length
**Run Average Speed**	General	Speed
**Right HL Body Speed**	General	Speed
**Left HL Body Speed**	General	Speed
**Phase Dispersion LF > RH**	Phase Dispersion	Diagonal
**Phase Dispersion RH > LF**	Phase Dispersion	Diagonal
**Phase Dispersion LH > RH**	Phase Dispersion	Contralateral
**Phase Dispersion RF > RH**	Phase Dispersion	Ipsilateral
**Phase Dispersion LF > LH**	Phase Dispersion	Ipsilateral

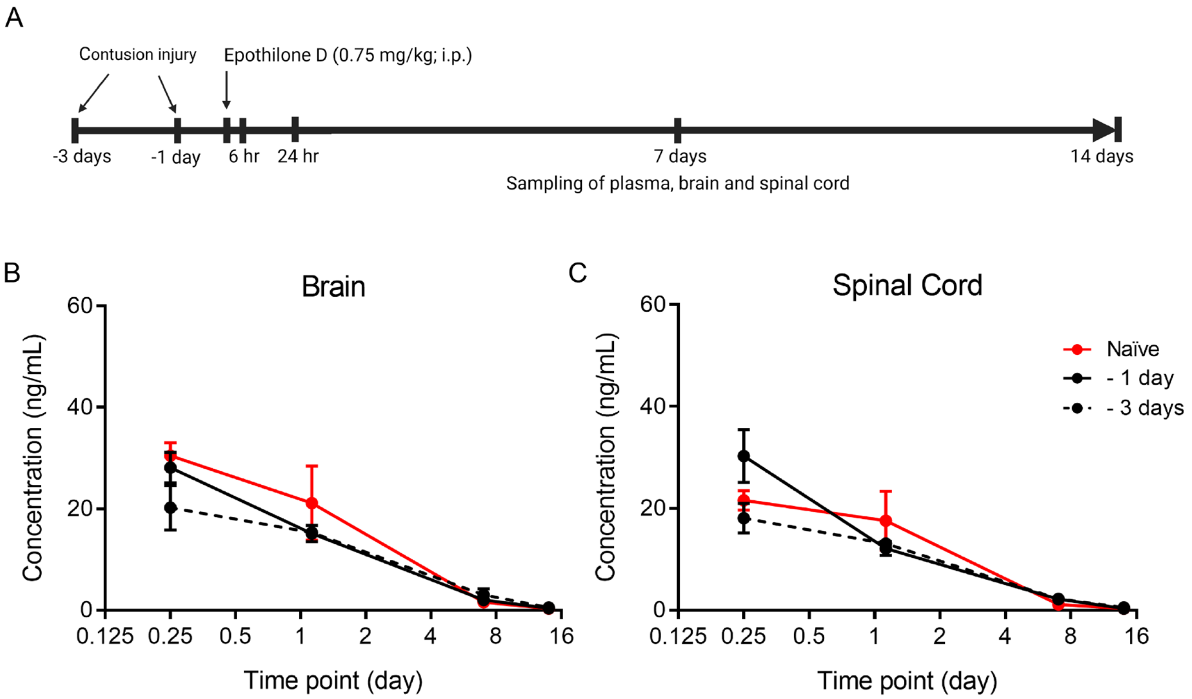

Supplementary Figure 1. Contusive spinal cord injury does not affect the pharmacokinetic profile of epothilone D. (A) Timeline of contusion injury, epoD injection and tissue sampling. **(B,C)** Concentrations of epothilone D in brain and spinal cord samples quantified by LC-MS following epoD injections in rats that received contusion injury three or one day prior to injection, or in naïve animals. Plotted data are mean drug concentration ± SEM. Two-way ANOVA and Bonferroni *post-hoc* where $*P < 0.05$. $n = 3$ animals per group and time point.

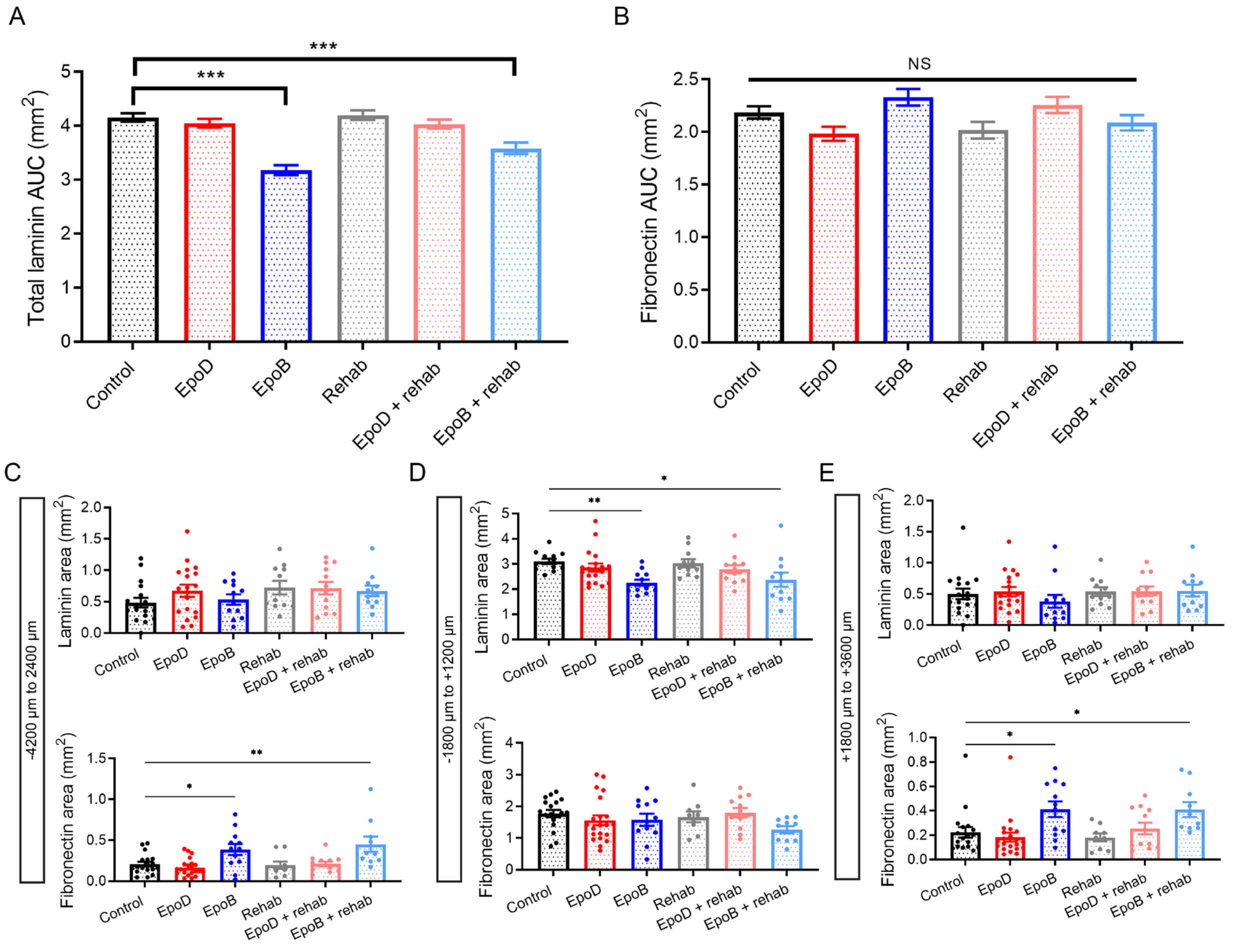

Supplementary Figure 2. Volumes of laminin and fibronectin expression throughout the lesion. (A) Total area under the curves of laminin (Control n = 16; epoD n = 18; epoB n = 12; rehabilitation n = 11; epoD + rehabilitation n = 12; epoB + rehabilitation n = 11) and fibronectin **(B)** immunoreactivity throughout the lesion (Control n = 19; epoD n = 20; epoB n = 12; rehabilitation n = 10; epoD + rehabilitation n = 12; epoB + rehabilitation n = 10). This expression was then segregated into regions **(C)** rostral to the lesion, **(D)** lesion center, **(E)** and caudal to the lesion. Plotted data are the mean ± SEM; (data points represents means per animal). *P < 0.05, **P < 0.01, ***P < 0.001 by two-way ANOVA and Bonferroni post-hoc.

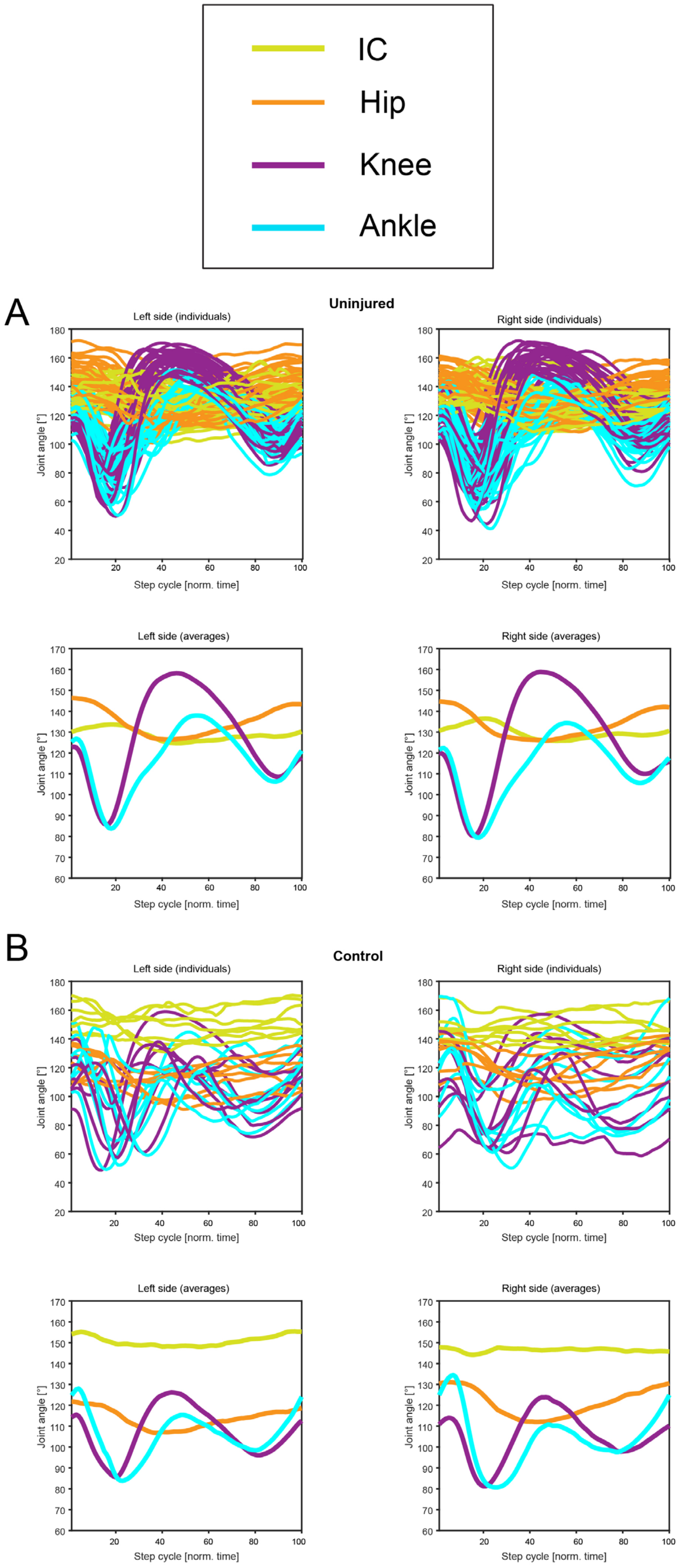
IC
Hip
Knee
Ankle
A
Uninjured
Left side (individuals)
Right side (individuals)
Left side (averages)
Right side (averages)
B
Control
Left side (individuals)
Right side (individuals)
Left side (averages)
Right side (averages)
Joint angle [°]
Step cycle [norm. time]

C

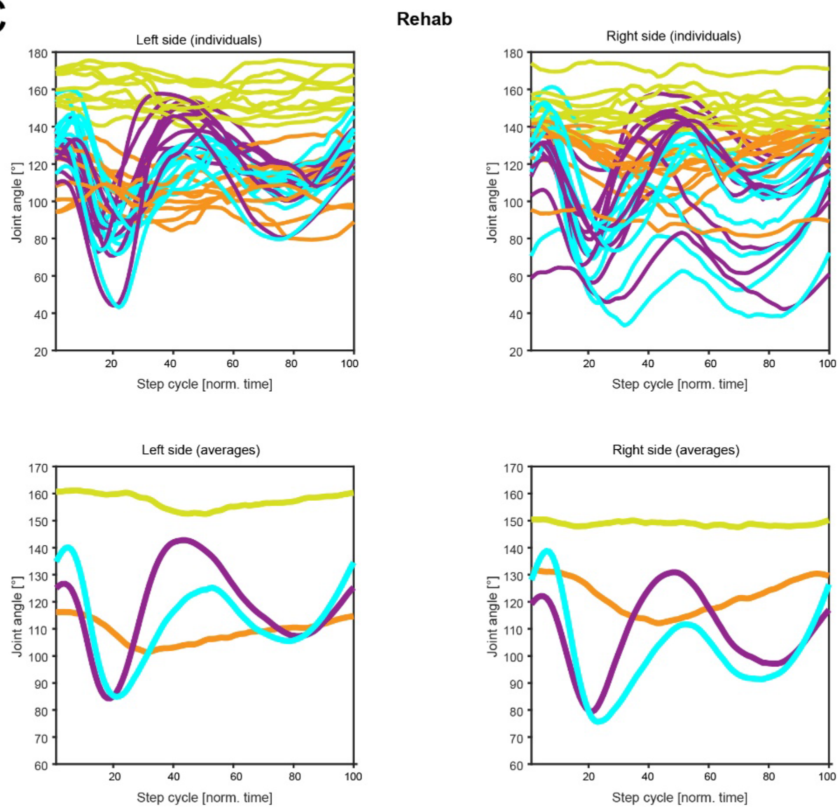

Rehab
Left side (individuals)
Right side (individuals)
Left side (averages)
Right side (averages)
Joint angle [°]
Step cycle [norm. time]

D

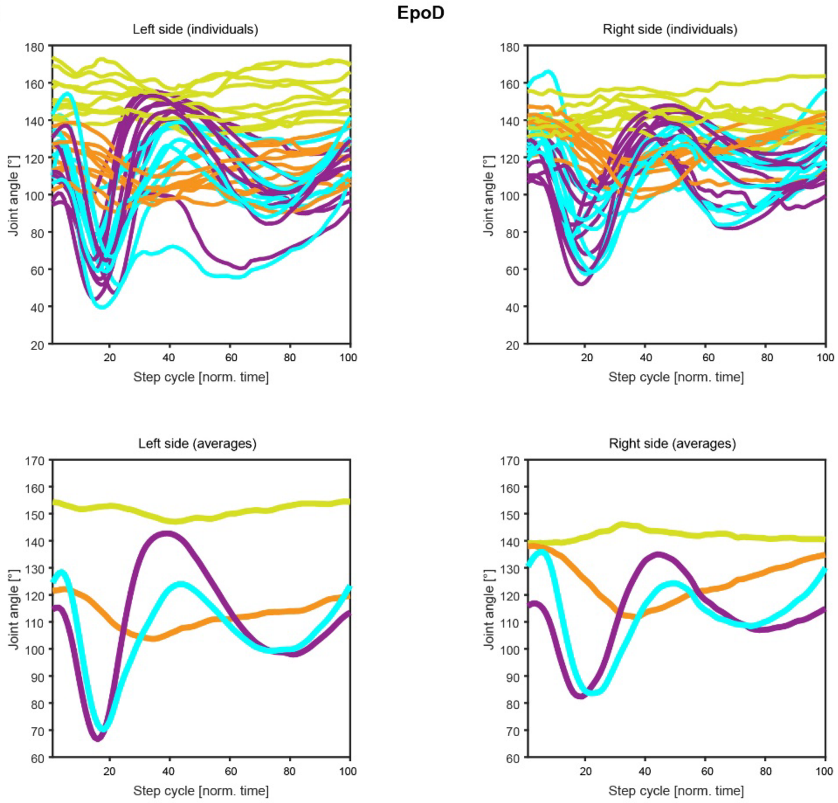

EpoD
Left side (individuals)
Right side (individuals)
Left side (averages)
Right side (averages)
Joint angle [°]
Step cycle [norm. time]

E

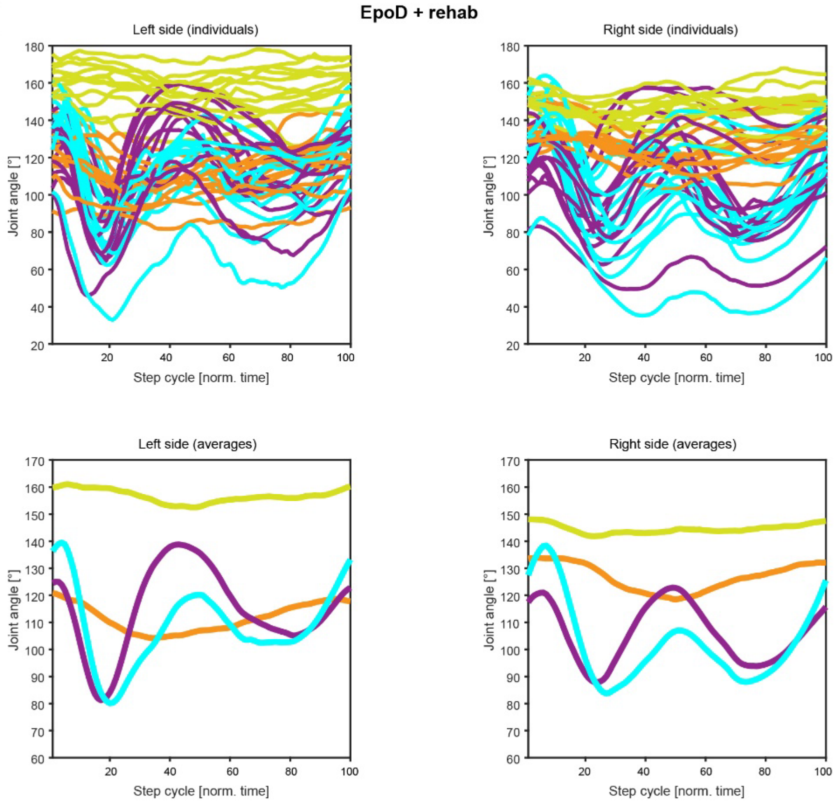

EpoD + rehab
Left side (individuals)
Right side (individuals)
Joint angle [°]
Step cycle [norm. time]
Left side (averages)
Right side (averages)

F

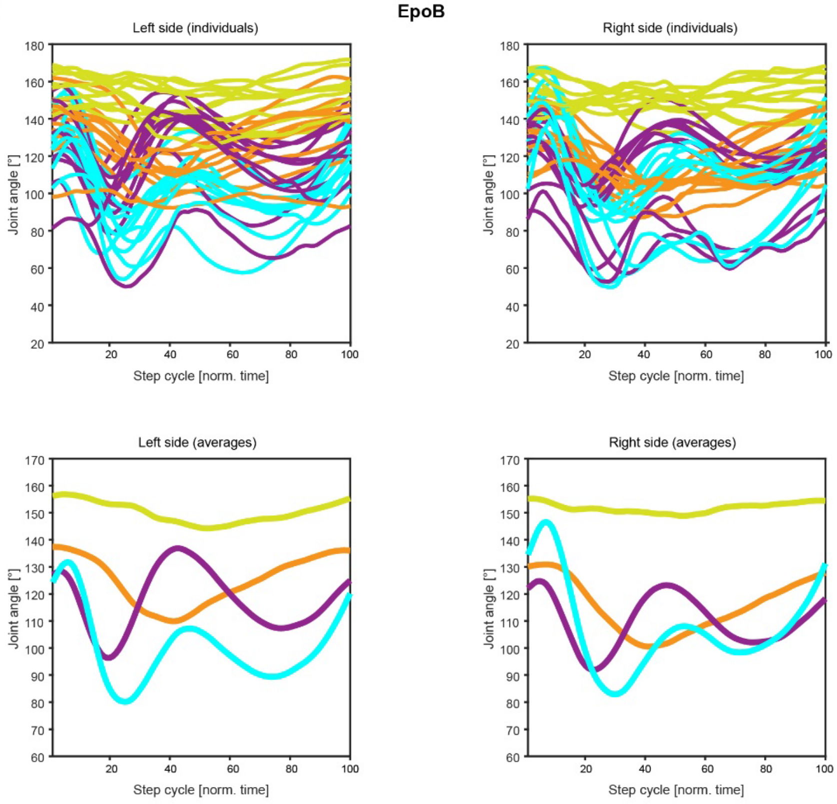

EpoB
Left side (individuals)
Right side (individuals)
Joint angle [°]
Step cycle [norm. time]
Left side (averages)
Right side (averages)

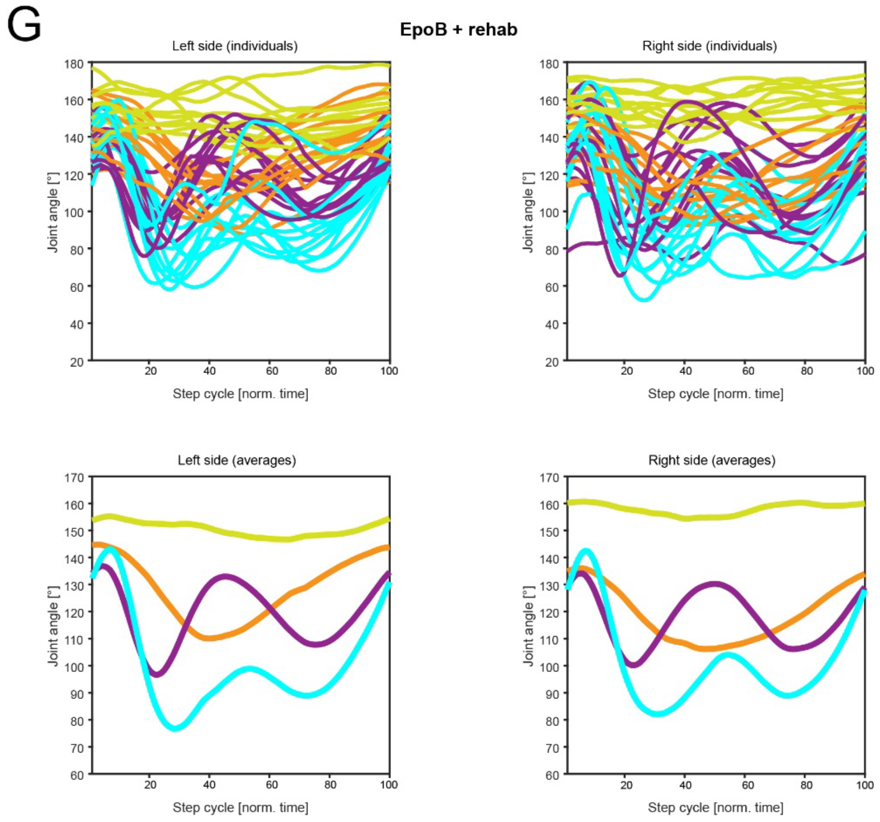

Supplementary Figure 3. Complete dataset of the joint angle time courses for all treatment groups and body sides. (A-G) Joint angle time courses (individual and averages) for all treatment groups and both left and right hindlimbs. All steps have been normalised to standard duration. Uninjured = 31; Control *n* = 9; epoD *n* = 10; epoB *n* = 11; rehabilitation *n* = 10; epoD + rehabilitation *n* = 12; epoB + rehabilitation *n* = 11.

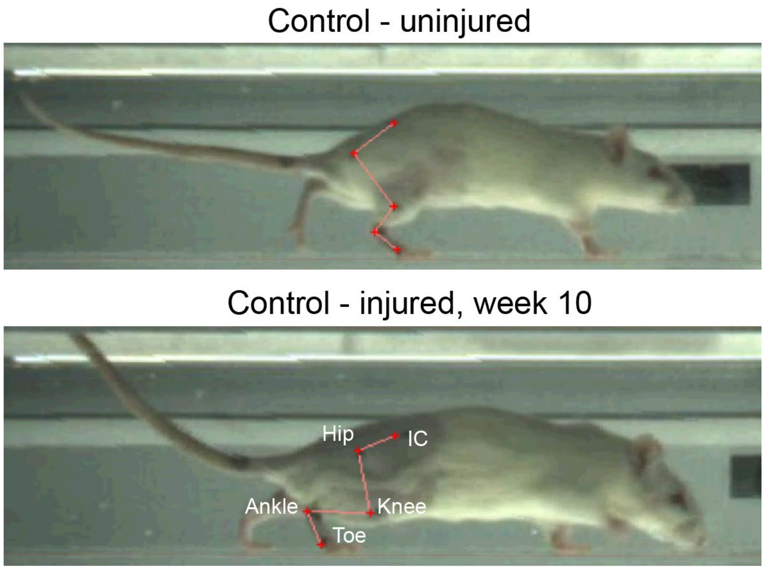

Supplementary Figure 4. Thoracic spinal cord injury results in a more crouched postured and decreased range of hindlimb motion. Representative frame from uninjured and injured control group animals during placement of the right paw.

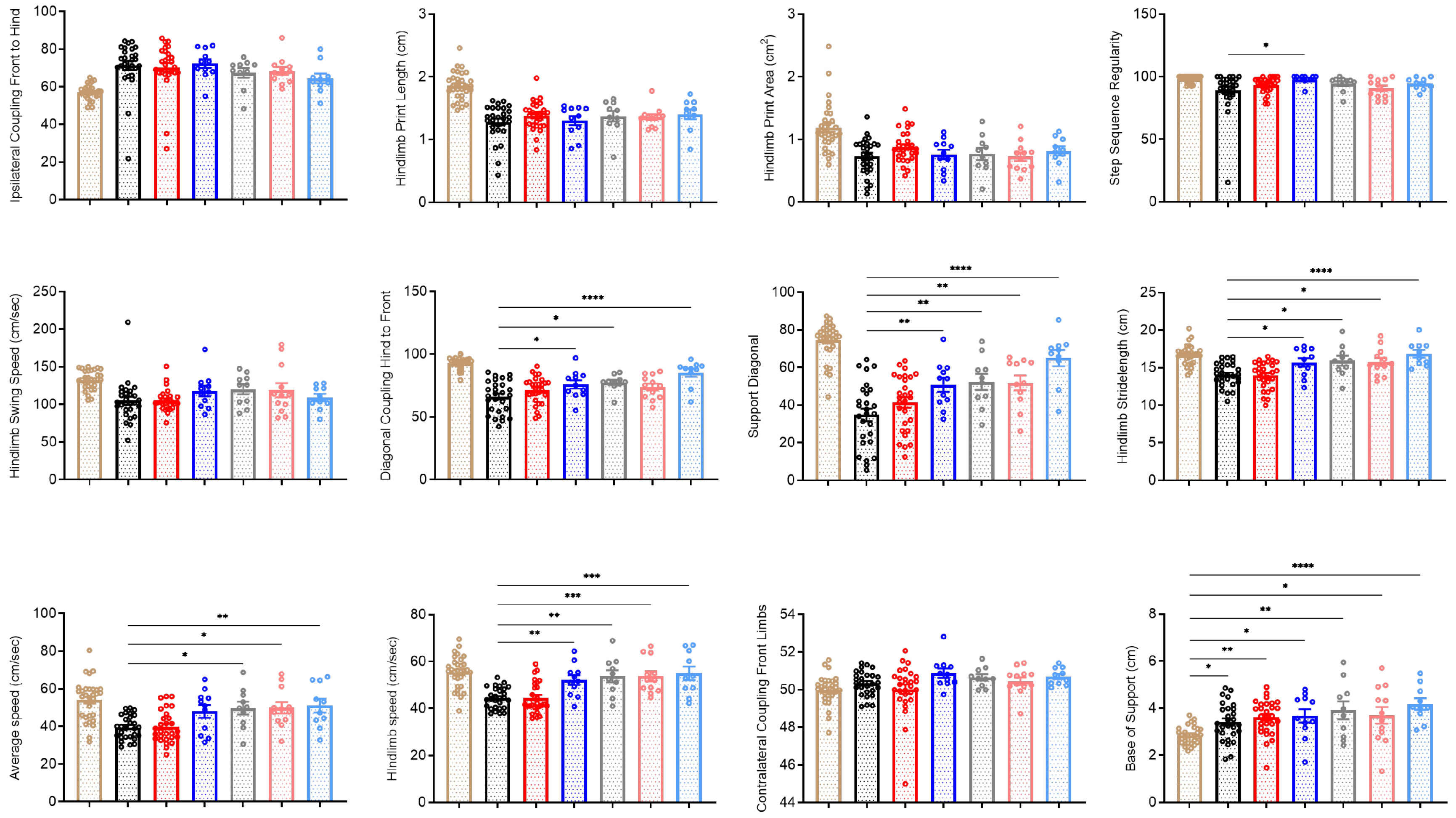

Ipsilateral Coupling Front to Hind
Hindlimb Print Length (cm)
Hindlimb Print Area (cm^2)
Step Sequence Regularity
Hindlimb Swing Speed (cm/sec)
Diagonal Coupling Hind to Front
Support Diagonal
Hindlimb Stridelength (cm)
Average speed (cm/sec)
Hindlimb speed (cm/sec)
Contralateral Coupling Front Limbs
Base of Support (cm)

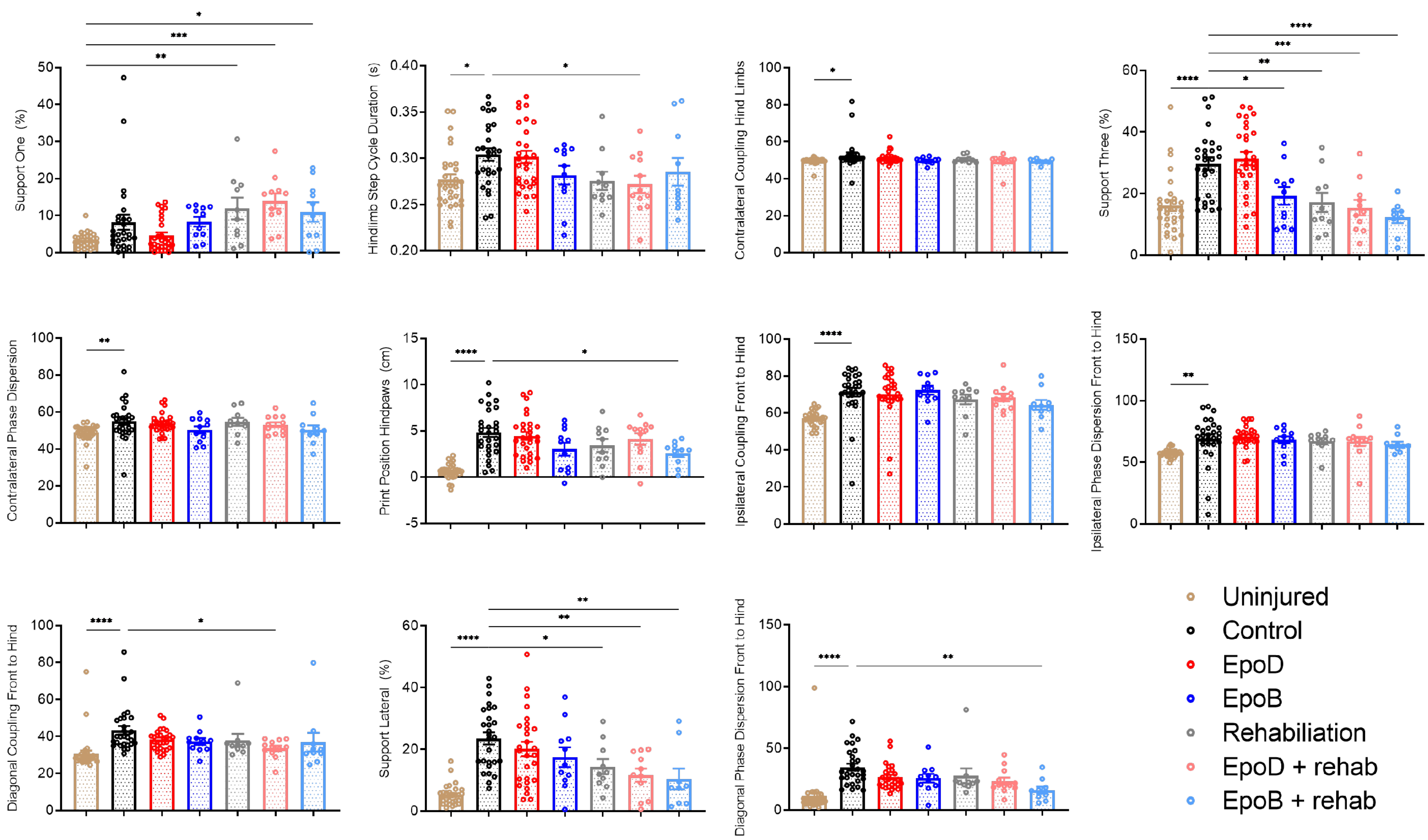
Support One (%)
Hindlimb Step Cycle Duration (s)
Contralateral Coupling Hind Limbs
Support Three (%)
Contralateral Phase Dispersion
Print Position Hindpaws (cm)
Ipsilateral Coupling Front to Hind
Ipsilateral Phase Dispersion Front to Hind
Diagonal Coupling Front to Hind
Support Lateral (%)
Diagonal Phase Dispersion Front to Hind
Uninjured
Control
EpoD
EpoB
Rehabiliation
EpoD + rehab
EpoB + rehab

Supplementary Figure 5. The individual graphs and parametric statistical analysis of the catwalk dataset. Individual graphs were plotted for each parameter used in the catwalk data analysis and presented in the order that they appear in Figure 7. Plotted data are the mean ± SEM; (data points represents means per animal). *$P < 0.05$, **$P < 0.01$, ***$P < 0.001$, ****$P < 0.0001$ by two-way ANOVA, Dunnett's *post-hoc*. Uninjured = 31; Control $n = 28$; epoD $n = 28$; epoB $n = 11$; rehabilitation $n = 10$; epoD + rehabilitation $n = 12$; epoB + rehabilitation $n = 10$.

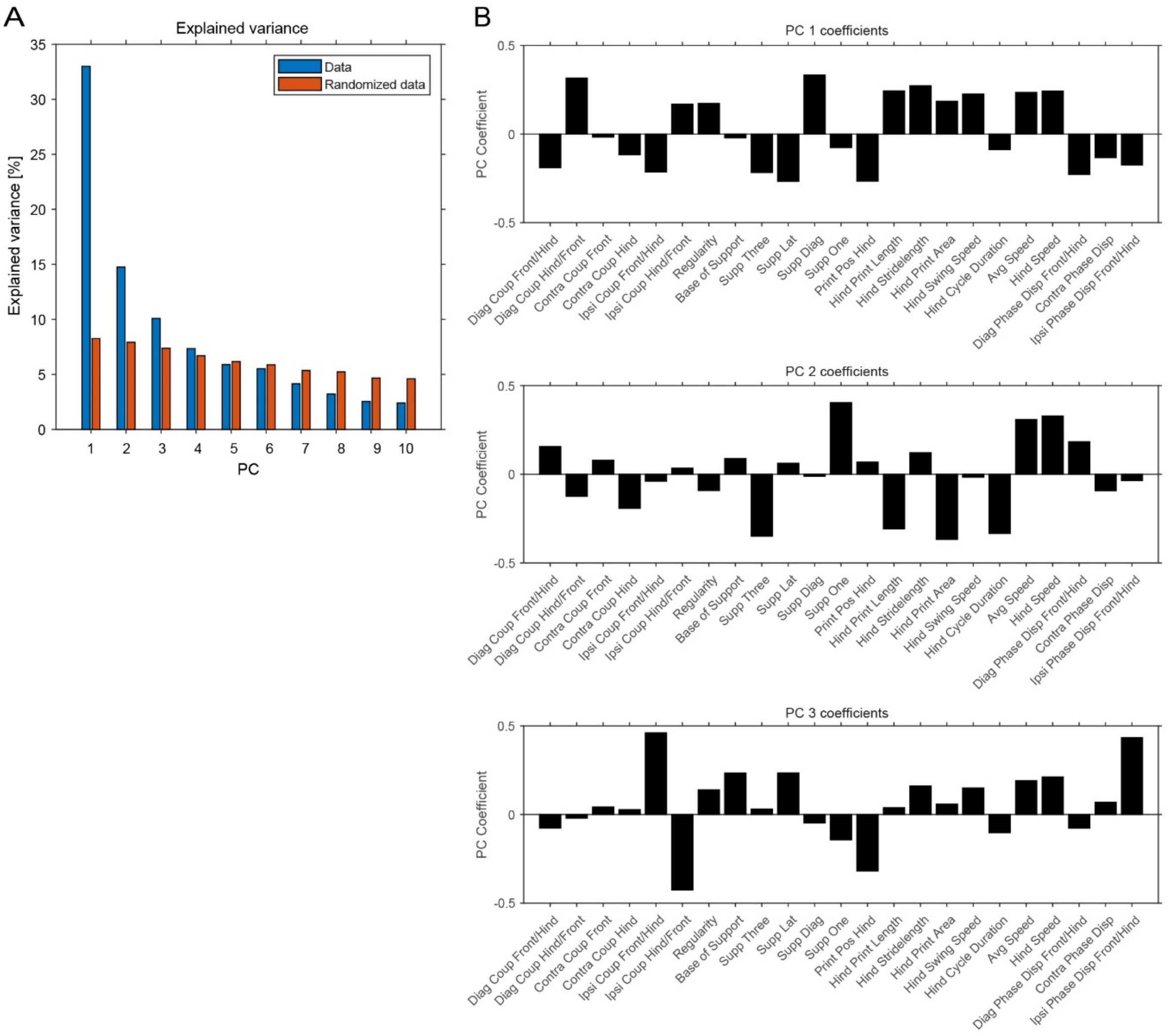

Supplementary Figure 6. Explained variance within the Catwalk PCA dataset and the PC coefficients of each parameter. (A) The explained variance of the first 10 PCs from the Catwalk data (blue bars) compared to variance generated from a random dataset (orange bars). **(B)** PC coefficients (or loadings) for the first three PCs and all measured parameters. Uninjured = 31; Control *n* = 28; epoD *n* = 28; epoB *n* = 11; rehabilitation *n* = 10; epoD + rehabilitation *n* = 12; epoB + rehabilitation *n* = 10.

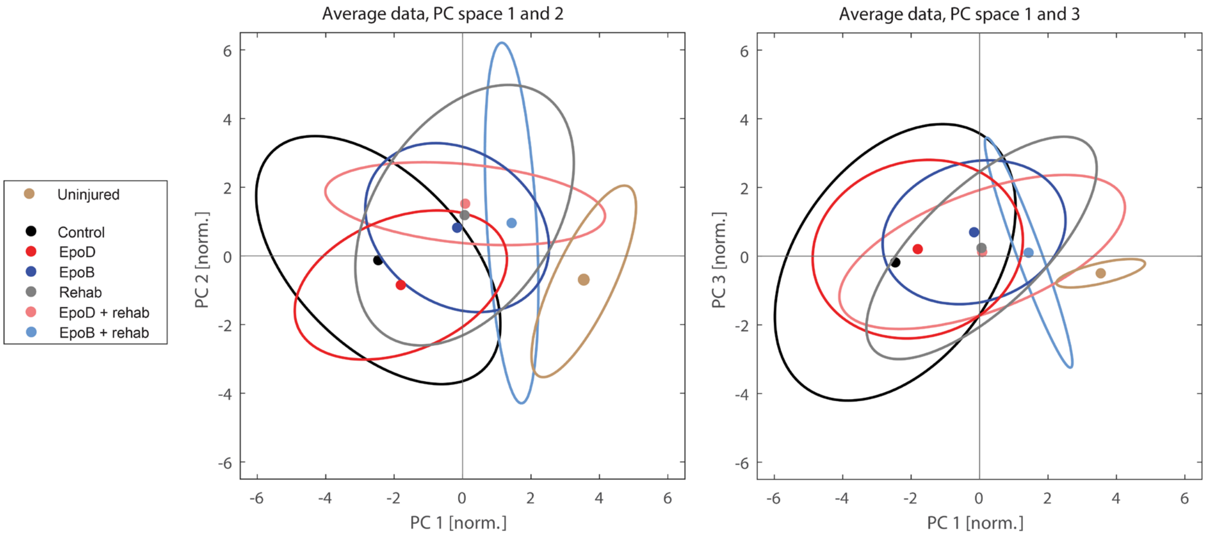

Supplementary Figure 7. Principal component score for the first three PC's. Complete (one standard deviation ellipses, first two panels) and average (dots, first two panels) data projected into the subspaces spanned by their first three PCs. Uninjured = 31; Control n = 28; epoD n = 28; epoB n = 11; rehabilitation n = 10; epoD + rehabilitation n = 12; epoB + rehabilitation n = 10.
